# Regulation of lysosomal trafficking of progranulin by sortilin and prosaposin

**DOI:** 10.1093/braincomms/fcab310

**Published:** 2022-01-04

**Authors:** Huan Du, Xiaolai Zhou, Tuancheng Feng, Fenghua Hu

**Affiliations:** Department of Molecular Biology and Genetics, Weill Institute for Cell and Molecular Biology, Cornell University, Ithaca, NY 14853, USA; Department of Molecular Biology and Genetics, Weill Institute for Cell and Molecular Biology, Cornell University, Ithaca, NY 14853, USA; State Key Laboratory of Ophthalmology, Zhongshan Ophthalmic Center, Sun Yat-sen University, Guangzhou, China; Department of Molecular Biology and Genetics, Weill Institute for Cell and Molecular Biology, Cornell University, Ithaca, NY 14853, USA; Department of Molecular Biology and Genetics, Weill Institute for Cell and Molecular Biology, Cornell University, Ithaca, NY 14853, USA

**Keywords:** frontotemporal lobar degeneration, progranulin, prosaposin, sortilin, lysosome

## Abstract

Haploinsufficiency of the progranulin protein is a leading cause of frontotemporal lobar degeneration. Accumulating evidence support a crucial role of progranulin in the lysosome. Progranulin comprises 7.5 granulin repeats and is known to traffic to lysosomes via direct interactions with prosaposin or sortilin. Within the lysosome, progranulin gets processed into granulin peptides. Here, we report that sortilin and prosaposin independently regulate lysosomal trafficking of progranulin *in vivo*. The deletion of either prosaposin or sortilin alone results in a significant decrease in the ratio of granulin peptides versus full-length progranulin in mouse brain lysates. This decrease is further augmented by the deficiency of both prosaposin and sortilin. A concomitant increase in the levels of secreted progranulin in the serum was observed. Interestingly, while the deletion of both prosaposin and sortilin totally abolishes lysosomal localization of progranulin in neurons, it has a limited effect on lysosomal trafficking of progranulin in microglia, suggesting the existence of a novel sortilin and prosaposin independent pathway mediating progranulin lysosomal trafficking. In summary, our studies shed light on the regulation of lysosomal trafficking and processing of progranulin *in vivo*.

## Teaser

Du *et al*. report that the frontotemporal dementia protein progranulin traffics to the lysosome via two independent pathways, through binding to either prosaposin or sortilin in neurons. However, progranulin remains lysosome localized in microglia with both prosaposin and sortilin ablated, suggesting the existence of an alternative trafficking mechanism.

## Graphical Abstract

**Graphical Abstract:**
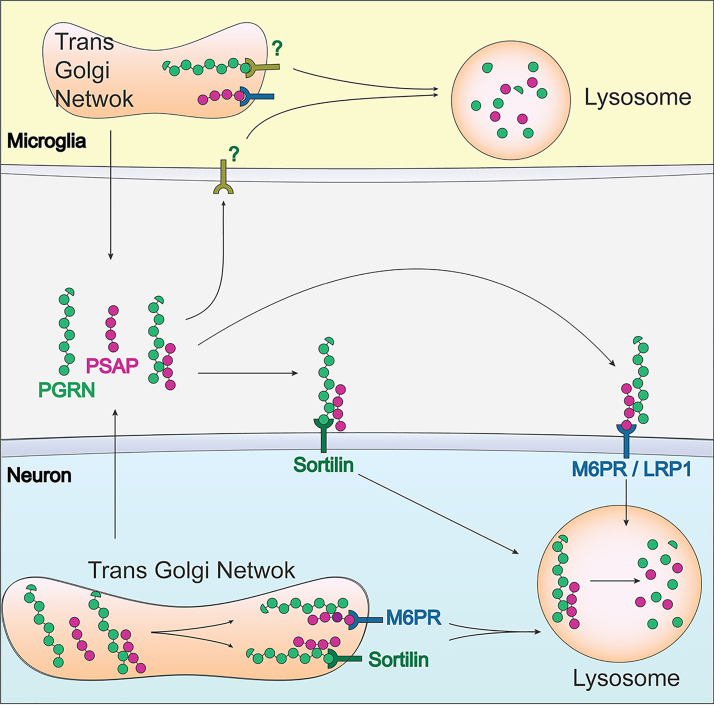

## Introduction

Frontotemporal lobar degeneration (FTLD) is a devastating neurodegenerative disease which affects ∼250 000 people in the USA.^1,2^ Heterozygous mutations in the *granulin (GRN)* gene, resulting in progranulin (PGRN) haploinsufficiency, are one of the major causes of FTLD with TDP-43 and ubiquitin-positive inclusions (FTLD-TDP).^3–5^ PGRN is an evolutionarily conserved, secreted glycoprotein of 88 kDa, comprising 7.5 granulin modules.^6–10^ The neurotrophic and anti-inflammatory functions of PGRN and granulin peptides are thought to prevent neurodegeneration in the ageing brain.^9,11–21^ However, multiple recent studies have suggested a critical role of PGRN in the lysosome.^22^ First, homozygous PGRN mutant human patients exhibit neuronal ceroid lipofuscinosis (NCL), a lysosomal storage disorder.^23,24^ PGRN knockout mice also accumulate the lysosomal byproduct, lipofuscin.^17^ Most importantly, NCL-related phenotypes are reported in FTLD patients with *GRN* mutation,^25–27^ supporting the theory that lysosomal dysfunction might serve as a common pathogenetic mechanism of these two diseases. Secondly, PGRN is transcriptionally co-regulated with a number of essential lysosomal genes by the transcriptional factor TFEB, a master regulator of lysosomal biogenesis.^28,29^ Finally, PGRN has been shown to be a lysosome resident protein and there are two independent pathways for PGRN lysosome trafficking. Sortilin, a trafficking receptor of the vacuolar protein sorting 10 (VPS10) family, was shown to interact with PGRN and traffic PGRN to the lysosome.^30^ In addition, PGRN interacts with another lysosomal protein prosaposin (PSAP) to get a ‘piggy-back’ ride to the lysosome through the PSAP receptors mannose 6-phosphate receptor (M6PR) and a low-density lipoprotein receptor-related protein 1 (LRP1) in a sortilin independent manner.^31^ In the lysosome, PSAP is processed to saposin peptides, which regulate enzymes involved in glycosphingolipid degradation.^32^ Interestingly, recently several studies have shown that PGRN is processed in a similar manner to individual granulin peptides in the lysosome and several proteases, including cathepsin B, L and D have been implicated in the process.^33–35^ The granulin peptides have been shown to regulate the activities of several lysosome enzymes, including cathepsin D^27,36–38^ and glucocerebrosidase.^39–41^ Furthermore, heterozygous mutations in the *GRN* gene not only leads to the haploinsufficiency of full-length PGRN but also decreased levels of granulin peptides.^33^

Anti-sortilin antibodies which block the PGRN–sortilin interaction are currently in clinical trials attempting to boost circulating PGRN levels to treat FTLD patients with *GRN* mutations (NCT04111666, NCT04374136, NCT03987295). Thus, it is important to examine the effect of sortilin ablation on PGRN trafficking and processing *in vivo*. In this study, we generated mice deficient in both PSAP and sortilin and analysed PGRN lysosome localization, the levels of granulin peptides as well as serum PGRN levels. Our results further confirm that PSAP and sortilin are two independent lysosome trafficking pathways for PGRN and support the existence of an additional lysosomal trafficking pathway of PGRN in microglia.

## Materials and methods

### Primary antibodies and reagents

The following antibodies were used in this study: sheep anti-PGRN and granulin peptides (R&D Systems, AF2557) (1:750 for western blot, 1:150 for immunofluorescence staining), mouse anti-GAPDH (Proteintech Group, 60004-1-Ig), rat anti-mouse LAMP1 (BD Biosciences, 553792), rabbit anti IBA-1 (Wako, 01919741), goat anti-sortilin (Novus Biologicals, NB100-1028) and rabbit anti-PSAP antibodies as previouls described.^31^

The following reagents were also used in the study: Dulbecco’s modified Eagle’s medium (DMEM) (Cellgro, 10-017-CV), 0.25% Trypsin (Corning, 25-053-CI), Odyssey blocking buffer (LI-COR Biosciences, 927-40000), TrueBlack Lipofuscin Autofluorescence Quencher (Biotium, 23007), protease inhibitor (Roche, 05056489001), Pierce BCA Protein Assay Kit (Thermo scientific, 23225), O.C.T compound (Electron Microscopy Sciences, 62550-01) and mouse PGRN ELISA Kit (BioLegend, 430901).

### Mouse strains

C57/BL6 and *Grn*^−/−42^ mice were obtained from the Jackson Laboratory. Sortilin knockout mice^43^ were a gift from S. Strittmatter (Yale University, New Haven, CT, USA) and A. Nykjaer (Aarhus University, Aarhus, Denmark). *Psap* knockout mice were previously described^44^  *Psap^+/^*^−^ and *Sort*^−/−^ mice was mated to generate *Psap^+/^*^−^  *Sort^+/^*^−^ mice; *Psap^+/^*^−^  *Sort^+/^*^−^ mice were bred with each other to generate *Psap^+/^*^−^  *Sort*^−/−^ mice, which were mated to give *Psap*^−/−^  *Sort*^−/−^ mice. Sortilin genotyping was performed using the following primers: Forward wild-type (WT), 5′-AAACAATCCTTCCATACCCAC-3′; Reverse WT, 5′-CCCCTTGTATTTCCTGTGGAC-3′; Reverse sortilin knockout, 5′-GATTGGGAAGACAATAGCAGG-3′ (850 bp WT, 600 bp *Sort*^−/−^). *Psap*genotyping was performed using the following primers 5′-TTCAGCAAGTTCCCAGCTTCGG-3′; 5′-GAGCCCAATTTTAGCAAGAGA-3′ (312 bp WT, 1500 bp *Psap*^−/−^). The age of the mice used was described in the figure legend for each experiment. Both male and female mice were used and the sex of the mice in each experiment was matched in the same experiment. *Psap*^−/−^ and *Psap*^−/−^  *Sort^+/^*^−^ mice were collected at weaning age (P21) when they showed severe behavioural deficits. All the mice were housed in the Weill Hall animal facility at Cornell. All animal procedures have been approved by the Institutional Animal Care and Use Committee (IACUC) at Cornell.

### Cell culture and biochemical assays

BV2 cells were maintained in Dulbecco’s Modified Eagle’s Medium (Cellgro) supplemented with 10% foetal bovine serum (Sigma) in a humidified incubator at 37°C with 5% CO_2_. To generate CRISPR constructs against mouse *Psap*, two oligonucleotides with the sequences 5′-CACCGAAGAGGGCGAGGGCGTACA-3′ and 5′-AAACTGTACGCCCTCGCCCTCTTC-3′ were annealed and ligated to pLenti-CRISPRv2 (Addgene). Lentiviruses were generated by transfecting HEK293T cells with pLenti-CRISPRv2 constructs together with pMD2.G and psPAX2 plasmids. For CRISPR-mediated genome editing, BV2 cells were infected with lentiviruses containing pLenti-CRISPRv2 harbouring guide RNA sequences targeted to mouse *Psap*. The cells were selected with puromycin (2 µg/ml) 7 days after infection and the knockout is confirmed by western blot and immunostaining.

### Western blot analysis

Mice were perfused with phosphate-buffered saline (PBS) and the tissues were dissected and snap-frozen with liquid nitrogen and kept at −80°C. On the day of the experiment, the frozen tissues were thawed and homogenized on the ice with bead homogenizer (Moni International) in ice-cold RIPA buffer [150 mM NaCl, 50 mM Tris–HCl (pH 8.0), 1% Triton X-100, 0.5% sodium deoxycholate, 0.1% sodium dodecyl sulfate] with 1 mM phenylmethylsulfonyl fluoride, and 1× protease inhibitors (Roche). After centrifugation at 14 000 × *g* for 15 min at 4°C, supernatants were collected. Protein concentrations were determined via BCA assay, and then standardized. Samples were separated by 4–12% Bis-Tris PAGE (Invitrogen) and transferred to 0.2 µm nitrocellulose. Membranes were blocked with LiCor Odyssey blocking buffer for 2 h at room temperature followed by incubation with primary antibody overnight at 4°C with gentle rocking. Membranes were then washed with Tris-buffered saline with 0.1% Tween-20 (TBST) three times, 10 min each, and incubated with fluorescently tagged secondary antibodies (LI-COR Biosciences) for 1 h at room temperature, followed by three washes. Membranes were scanned using an Odyssey Infrared Imaging System (LI-COR Biosciences). Densitometry was performed using Image Studio (LI-COR Biosciences) and Image J.

### Immunofluorescence staining, image acquisition and analysis

For brain section staining, mice were perfused with cold PBS and tissues were post-fixed with 4% paraformaldehyde. After dehydration in 30% sucrose buffer, tissues were embedded in O.C.T compound (Electron Microscopy Sciences). Twenty-micrometre-thick brain sections were cut with cryotome. Tissue sections were blocked and permeabilized with 0.1% saponin in Odyssey blocking buffer before incubating with primary antibodies overnight at 4°C. The next day, sections were washed 3× with cold PBS followed by incubation with secondary fluorescent antibodies and Hoechst at room temperature for 1 h. The slides were then mounted using mounting medium (Vector Laboratories). To block the autofluorescence in aged mice, brain sections from 12-month-old mice were incubated with 1× TrueBlack Lipofuscin Autofluorescence Quencher (Biotium) in 70% ethanol for 30 s at room temperature after the staining process. Images were acquired on a CSU-X spinning disc confocal microscope (Intelligent Imaging Innovations) with an HQ2 CCD camera (Photometrics) using 100× objectives, 10–12 different random images were captured. Lower magnification images were captured by 20× objectives on a Leica DMi8 inverted microscope, three to five images were captured from each sample. Data from ≥3 brains in each genotype were used for quantitative analysis.

For the quantitative analysis of neuronal PGRN levels in the brain sections, first neurons were selected based on the size of nuclei and PGRN expression, then the fluorescence intensity and area were measured directly using ImageJ after a threshold application. To quantify microglia PGRN levels in the brain sections, microglia was selected based on microglia maker IBA1 staining and the images were analysed using the same procedure in ImageJ. The total fluorescence signals were quantified. To quantify the degree of colocalization between PGRN and the lysosomal marker LAMP1, the JACoP plugin was used to generate Manders’ overlap coefficients.^45^ Three brain sections per mouse, separated by 100 µm, were used for quantification. The mean from the three sections was used to be representative of each mouse. Data were normalized to age-matched controls.

### Reverse transcription-quantitative polymerase chain reaction

Mouse cortical tissue was dissected and frozen in liquid nitrogen. The total RNAs were extracted using TRIzol (Invitrogen) and purified with Quick RNA MiniPrep Kit (Zymo Research). One microgram of total RNA was reverse transcribed to cDNA using poly (T) primer and SuperScript III reverse transcriptase (Invitrogen). Quantitative polymerase chain reaction was performed on a Light-Cyler 480 (Roch Applied Science), and the transcripts levels were measured using efficiency-adjusted ΔΔ−CT. PGRN transcript was normalized to β-actin and TBP. The mouse PGRN primer pair sequences were 5′-ATGTGGGTCCTGATGAGCTG-3′ and 5′-GCTCGTTATTCTAGGCCATGTG-3′. Mouse β-actin primers were 5′-ACGAGGCCCAGAGCAAGAG-3′ and 5′-TCTCCAAGTCGTCCCAGTTG-3′. Mouse TBP primers were 5′-CCCCACAACTCTTCCATTCT-3′ and 5′-GCAGGAGTGATAGGGGTCAT-3′.

### Enzyme-linked immunosorbent assay

Mouse serum was collected and analysed using mouse PGRN ELISA kit (R&D Systems, Catalogue no. DY2557) according to the manufacturer’s instructions as previously described.^31^

### Statistical analysis

All statistical analyses were performed using GraphPad Prism 8. All data are presented as mean ± SEM. Statistical significance was assessed by unpaired Student’s *t*-test (for two groups comparison) or one-way ANOVA tests with Bonferroni’s multiple comparisons (for multiple comparisons). *P*-values of ≤0.05 were considered statistically significant. *P < 0.05; **P < 0.01; *** *P* < 0.001.

### Availability of data and material

All data have been included in the manuscript. Additional data are available upon request.

## Results

### PGRN trafficking and processing in sortilin-deficient mice

**Figure 1:**
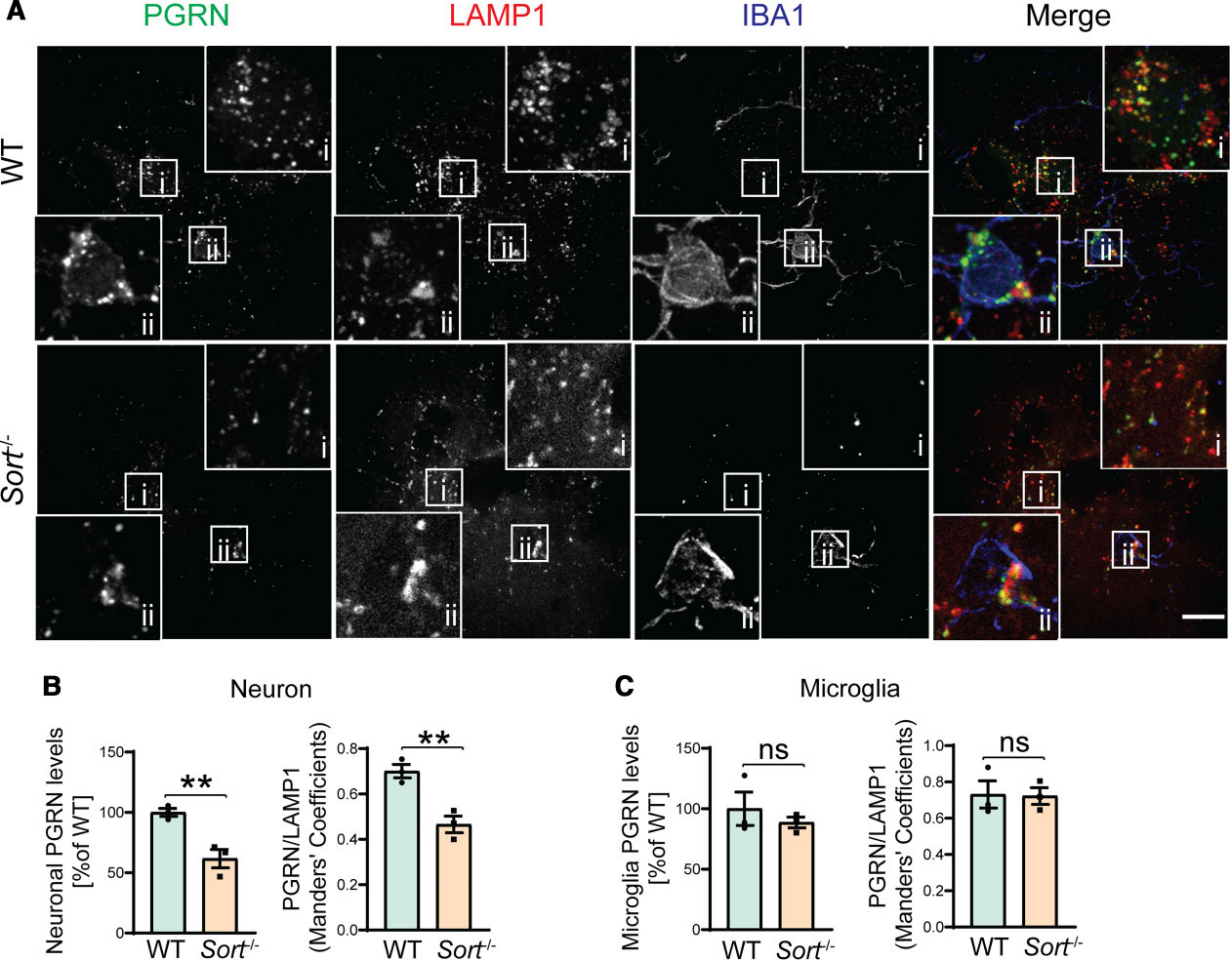
**PGRN levels are decreased in neurons in Sortilin-deficient mice**. (**A**) Brain sections of 3-month-old WT and *Sort*^−/−^ mice were stained with anti-PGRN, LAMP1 and IBA1 antibodies. Representative images for neurons and microglia in the cortex were shown in insets (i) and (ii), respectively. Scale bar, 10 µm. (**B)** Quantification of neuronal PGRN levels and overlap between PGRN and LAMP1 signals in neurons for experiment in **A**. Mean ± SEM; *n* = 3, Student’s *t*-test, **P < 0.01. (**C)** Quantification of microglial PGRN levels and overlap between PGRN and LAMP1 signals in microglia for experiment in **A**. Mean ± SEM; brain sections from three mice were analysed for each genotype (*n* = 3), Student’s *t*-test, ns, not significant.

Previously, we have shown that the ablation of sortilin in mice leads to an increase of PGRN levels in the brain lysates and in the serum.^30^ To examine the role of sortilin in PGRN trafficking more thoroughly, we stained the brain sections from age-matched WT and *Sort*^−/−^ mice with antibodies against PGRN, the main lysosomal membrane protein LAMP1 and microglia maker IBA1. Consistent with our previous findings, we found that neuronal signals of PGRN and more specifically, lysosomal pool of PGRN in neurons are significantly reduced by sortilin ablation in both 3- and 12-month-old mice (Fig. 1A and B and Supplementary Fig. S1). However, microglial PGRN remains localized in the lysosome compartment in *Sort*^−/−^ brain sections and microglial PGRN levels are not altered (Fig. 1A and C), which is consistent with low expression levels of sortilin in microglia compared to neurons.^30^

**Figure 2:**
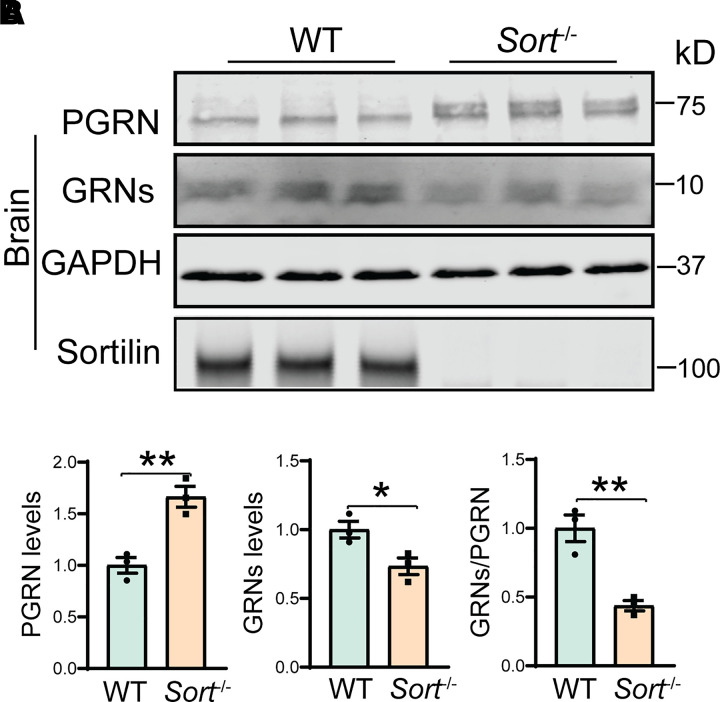
**PGRN processing in *Sort*^−/−^ mice.** (**A, B)** Western blot analysis of PGRN and granulin peptides in the cortical lysates from 3-month-old WT and *Sort*^−/−^ mice. The levels of PGRN and granulin peptides (GRNs) were quantified and normalized to GAPDH. Lysates from three mice were analysed for each genotype (*n* = 3), Student’s *t*-test, *P < 0.05; **P < 0.01.

Next, we examined the levels of granulin peptides and PGRN in the cortical lysates from 3-month-old WT and *Sort*^−/−^ mice by western blot analyses, using polyclonal antibodies that recognize both full-length PGRN and granulin peptides.^46^ Concomitant with an increase in the levels of full-length PGRN in *Sort*^−/−^ mice,^30^ a significant reduction in the levels of granulin peptides were observed in the cortical lysates from *Sort*^−/−^ mice, which results in a significant decrease in the ratio between granulin peptides and full-length PGRN (Fig. 2). Thus, lysosomal trafficking defects caused by sortilin deletion are associated with a decrease in PGRN processing.

### PGRN trafficking and processing in PSAP-deficient mice

**Figure 3:**
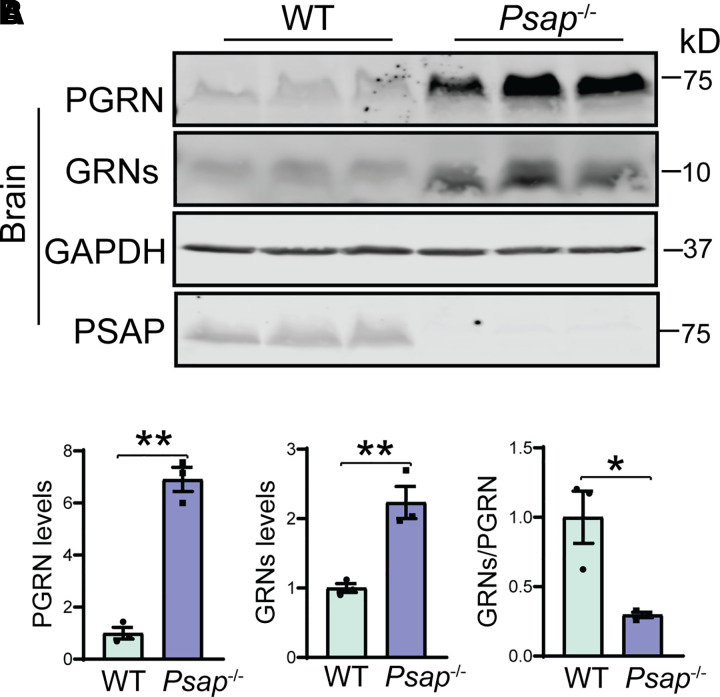
**PGRN processing in PSAP-deficient mice**. (**A, B)** Western blot analysis of PGRN and granulin peptides in the cortical lysates from P21 WT and *Psap*^−/−^ mice. The levels of PGRN and granulin peptides (GRNs) were quantified and normalized to GAPDH. Lysates from three mice were analysed for each genotype (*n* = 3), Student’s *t*-test, *P < 0.05, **P < 0.01.

Previously, we have shown that the PSAP interacts with PGRN and mediates sortilin independent lysosomal trafficking of PGRN.^31^ To further determine the role of PSAP in PGRN processing *in vivo*, we examined the levels of both granulin peptides and PGRN in the cortical lysates from postnatal day 21 (P21) WT and *Psap*^−/−^mice by western blot analyses, since *Psap*^−/−^ mice die around weaning age.^44^ The ablation of PSAP leads to severe lysosomal abnormalities,^31,44^ and thus, a significant increase in PGRN mRNA levels (Supplementary Fig. S2) and levels of both full-length PGRN and granulin peptides in the brain (Fig. 3). However, a significant decrease in the ratio between granulin peptides and full-length PGRN was observed, which supports that PSAP plays a critical role in regulating PGRN lysosomal trafficking *in vivo*.

### Ablation of PSAP and sortilin leads to defects in lysosomal trafficking and processing of PGRN

**Figure 4:**
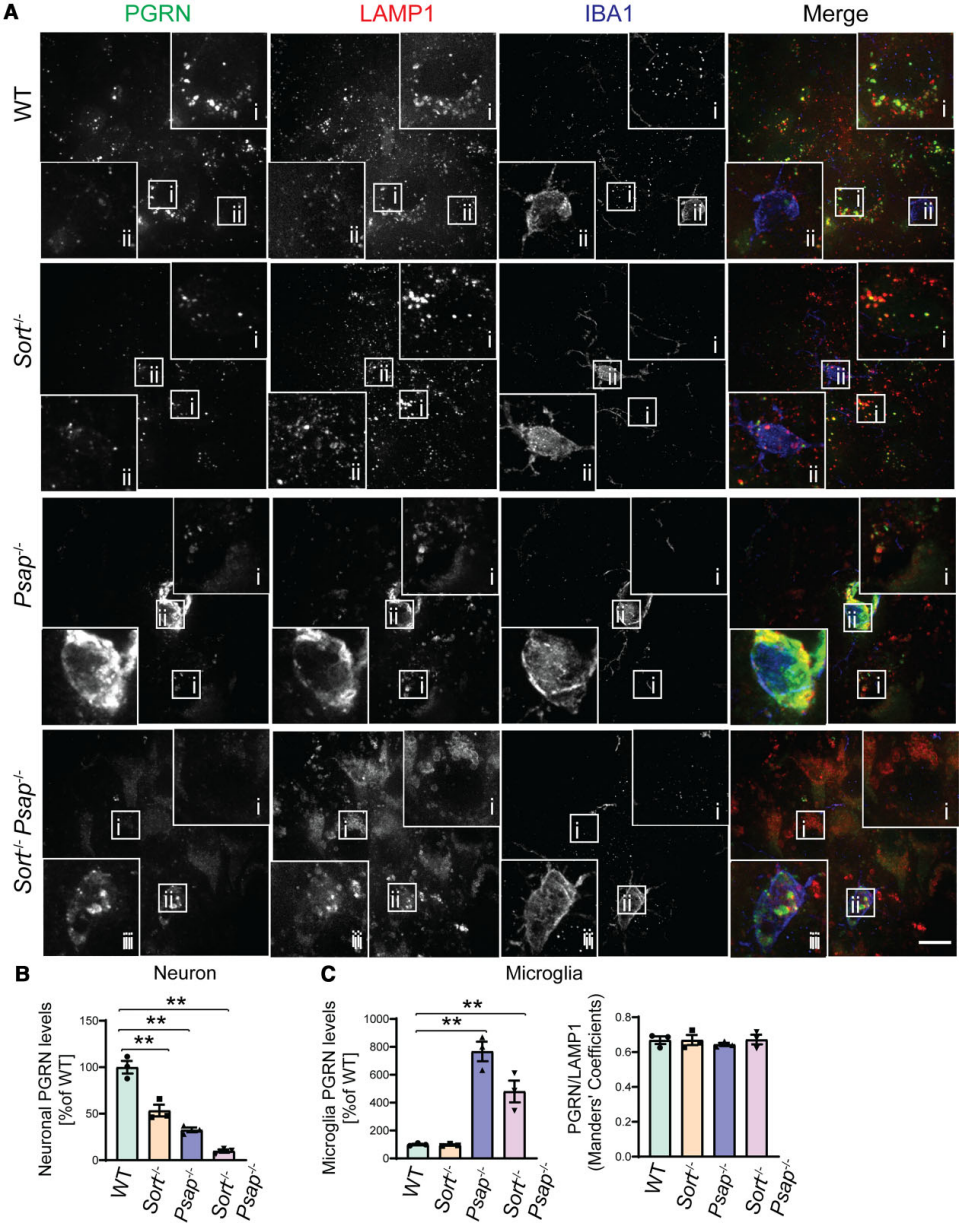
**Deficiency of both Sortilin and PSAP results in PGRN lysosomal trafficking defects in neurons but not in microglia.**. (**A)** Brain sections of P21 WT, *Psap*^−/−^*, Sort*^−/−^ and *Sort*^−/−^  *Psap*^−/−^ mice were stained with anti-PGRN, LAMP1 and IBA1 antibodies. A representative neuron from the cortex was shown in inset (i) and representative microglia was shown in inset (ii). Scale bar, 10 µm. (**B)** Quantification of neuronal PGRN levels in **A**. Mean ± SEM; *n* = 3, one-way ANOVA, **P < 0.01. (**C)** Quantification of microglial PGRN levels and overlap between PGRN and LAMP1 signals in microglia in **A**. Mean ± SEM; brain sections from three mice were analysed for each genotype (*n* = 3), one-way ANOVA, **P < 0.01.

**Figure 5:**
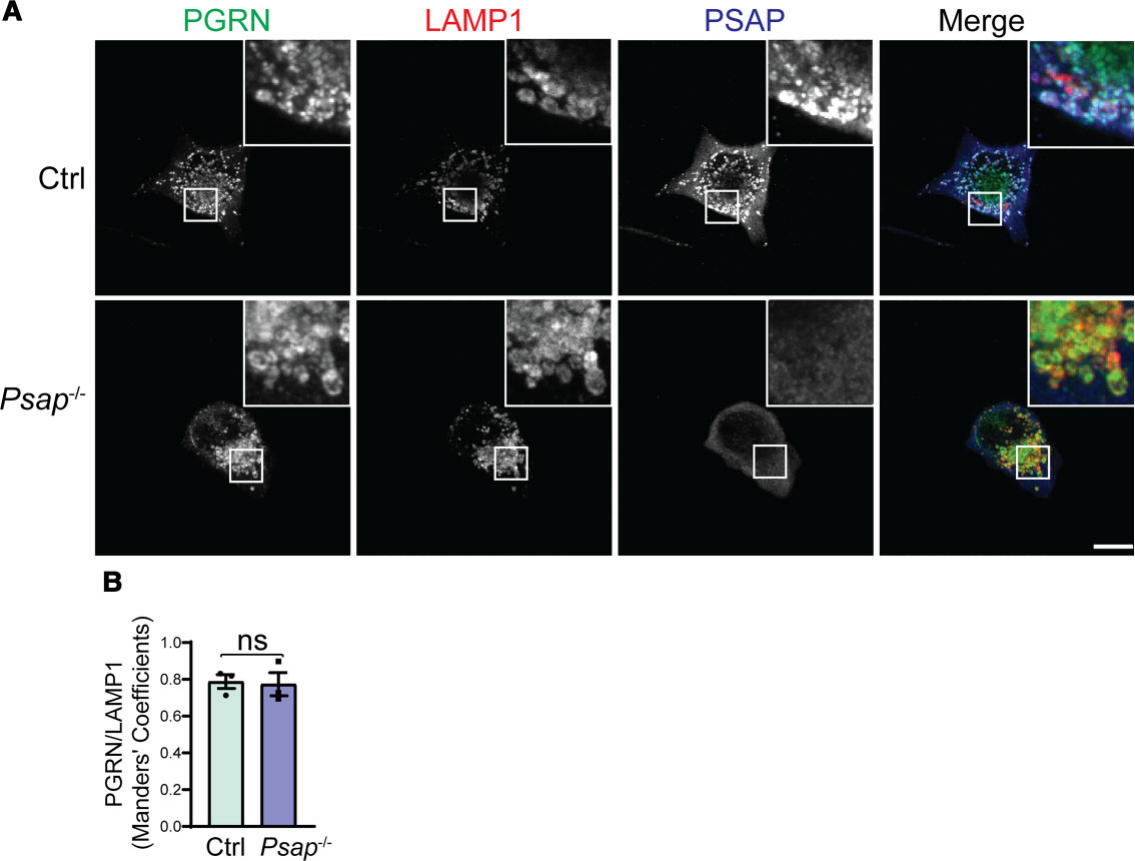
**PSAP ablation does not affect PGRN lysosomal trafficking in BV2 cells**. (**A)** Representative confocal images of BV2 cells stained with sheep anti-PGRN, rat anti-LAMP1 and rabbit anti-PSAP antibodies. Scale bar, 10 µm. (**B)** Quantification of PGRN signals inside LAMP1-positive vesicles in **A**. Mean ± SEM; *n* = 3, Student’s *t*-test, ns, not significant.

To further analyse the role of PSAP and sortilin in PGRN trafficking *in vivo*, we generated mice with both sortilin and PSAP ablated. Then we analysed the PGRN lysosomal localization in the brain sections from *Psap*^−/−^  *Sort*^−/−^ mice. The PGRN signals retained in the lysosome compartment in *Sort*^−/−^ neurons are completely lost in *Psap*^−*/*−^  *Sort*^−*/*−^ neurons (Fig. 4A and B), indicating that PSAP and sortilin are the only two pathways involved in the PGRN lysosome trafficking in neurons. Significant colocalization between PGRN and the lysosomal marker LAMP1 was observed in both *Psap*^−/−^ and *Psap*^−/−^  *Sort*^−/−^ microglia (Fig. 4A), indicating that there exists additional PSAP and sortilin independent PGRN lysosome trafficking mechanism in microglia. The increased intensity of PGRN was observed in both *Psap*^−/−^ and *Psap*^−/−^  *Sort*^−/−^ microglia due to the transcriptional up-regulation of PGRN (Supplementary Fig. S2). To further confirm this finding, we ablated PSAP in the microglial cell line BV2, which has very low levels of sortilin expression (Supplementary Fig. S3), and examined PGRN localization. PGRN remains localized in the lysosomal compartment in PSAP-deficient BV2 cells (Fig. 5A and B), further supporting that PGRN lysosomal trafficking in microglia is sortilin and PSAP independent.

**Figure 6:**
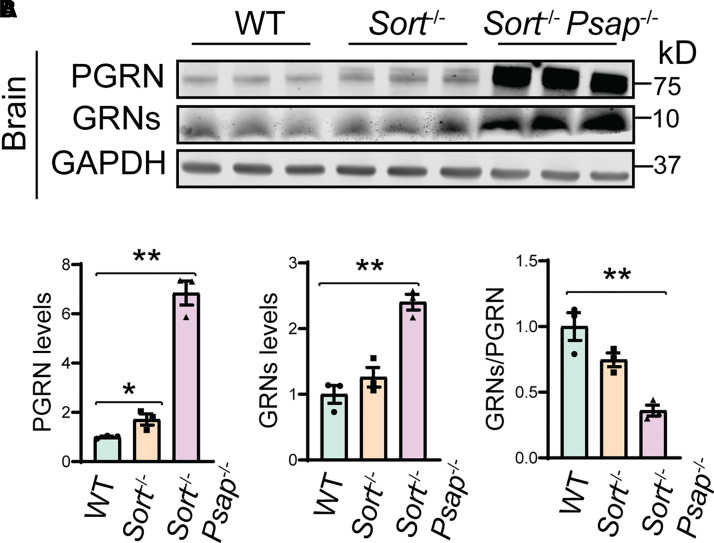
**PGRN processing in Sortilin- and PSAP-deficient mice.** (**A,B)** Western blot analysis of PGRN and granulin peptides in the cortical lysates from P21 WT, *Sort*^−/−^ and *Sort*^−/−^  *Psap*^−/−^ mice. The levels of PGRN and granulin peptides (GRNs) were quantified and normalized to GAPDH. Lysates from three mice were analysed for each genotype (*n* = 3), one-way ANOVA, *P < 0.05; **P < 0.01.

Next we examined the levels of granulin peptides in the cortical lysates from 3-week-old WT, *Sort*^−/−^*, Psap*^−/−^  *Sort*^−/−^ mice as another readout of PGRN lysosomal trafficking. In the 3-week-old mice, the ablation of sortilin results in a slight increase in the levels of both full-length PGRN and granulin peptides. A slight decrease in the ratio between the granulin peptides and PGRN was also observed (Fig. 6). Deficiency of both PSAP and sortilin results in an obvious increase in the levels of both PGRN and granulin peptides in the cortical lysates, with a significant decrease in the ratio between granulin peptides and full-length PGRN (Fig. 6). However, there are still significant amount of granulin peptides present in *Psap*^−/−^  *Sort*^−/−^ cortical lysates (Fig. 6A), consistent with our immunostaining results that PGRN lysosomal trafficking is affected in neurons but not microglia in *Psap*^−/−^  *Sort*^−/−^ mice (Fig. 4). Taken together, these data support that PSAP and sortilin serve as two independent lysosome trafficking pathways for PGRN *in vivo* and are the main two pathways to deliver PGRN to lysosomes in neurons, but there exist other sortilin and PSAP independent mechanisms to mediate PGRN lysosome trafficking in microglia, and possibly other cell types as well.

**Figure 7:**
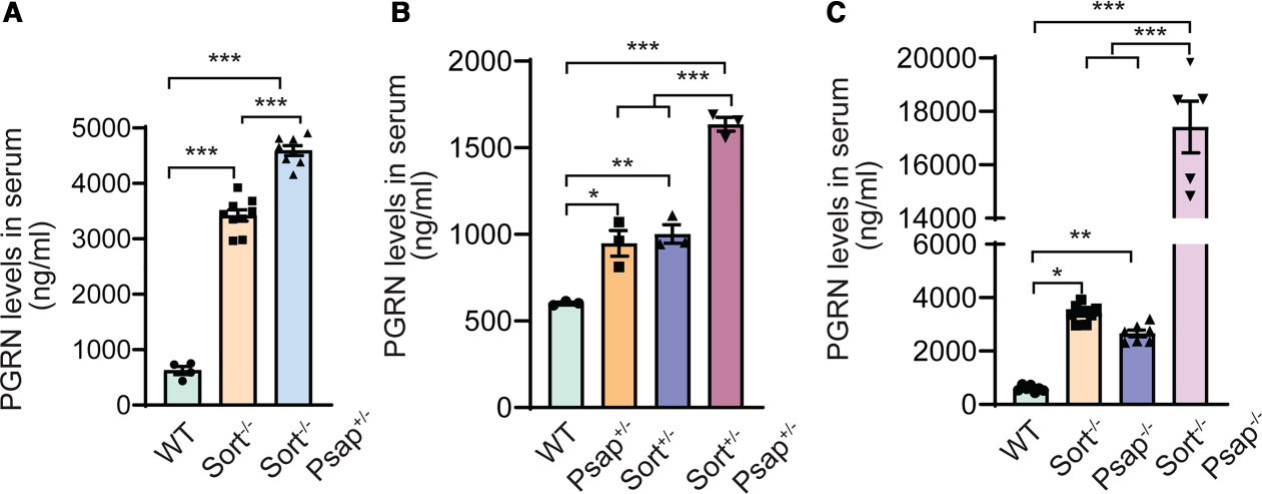
**Regulation of serum PGRN levels by Sortilin and PSAP**. **(A–C)** Serum PGRN levels in mice of different genotypes as indicated. Mean ± SEM; sera from three to nine mice were analysed for each genotype (*n* = 3–9), one-way ANOVA, *P < 0.05, **P < 0.01, ***P < 0.001.

Disruption of PGRN lysosome trafficking also results in increased sorting of PGRN to the secretory pathway and thus an increase in the serum levels of PGRN.^30,31^ To further analyse the role of PSAP and sortilin in PGRN trafficking *in vivo*, we measured the levels of secreted PGRN in the serum. In the WT mice, the concentration of PGRN in the serum is ∼500 ng/ml, which is increased to ∼3200 ng/ml in *Sort*^−/−^ mice. Deletion of one copy of *Psap* in *Sort*^−/−^ background (*Psap^+/^*^−^  *Sort*^−/−^) leads to a further increase in PGRN levels to 4500 ng/ml, indicating that PSAP haploinsufficiency exacerbates PGRN lysosome trafficking defects (Fig. 7A). Similarly, haploinsuffiency of both PSAP and Sortilin in *Psap^+/−^ Sort^+/−^* mice leads to a further increase in serum PGRN levels compared to *Psap^+/^*^−^ or *Sort^+/^*^−^ mice (Fig. 7B). In addition, ablation of both PSAP and sortilin leads to an increase of PGRN levels in the serum to ∼17 000 ng/ml, which is much more than in *Psap*^−/−^ mice (∼2500 ng/ml) or *Sort*^−/−^ mice (∼3200 ng/ml) (Fig. 7C), further confirming that PSAP and sortilin are two independent lysosome trafficking pathways for PGRN *in vivo*.

## Discussion

Previously, we have shown that PSAP and sortilin are two independent pathways mediating PGRN lysosomal trafficking.^31,47^ This is further supported by our analyses of PGRN lysosomal localization and PGRN processing in *Psap*^−/−^*, Sort*^−/−^ and *Psap*^−/−^  *Sort*^−/−^ mice. While deletion of sortilin or PSAP alone results in partial defect of PGRN lysosomal trafficking, ablation of both PSAP and sortilin leads to nearly complete loss of lysosomal PGRN signals in neurons. However, PGRN remain localized in the lysosome in *Psap*^−/−^  *Sort*^−/−^ microglia. In addition, the PSAP deletion in BV2 microglial cell line, which expresses very low levels of sortilin (Supplementary Fig. S3), does not affect lysosomal trafficking of PGRN. All these data support that while PSAP and sortilin are the two main pathways mediating PGRN lysosomal trafficking in neurons, there exist additional PSAP and sortilin independent mechanisms in microglia and possibly the other cell types to facilitate PGRN lysosomal delivery. It remains to be determined whether PGRN binds to a distinct lysosomal trafficking receptor or gets a ‘piggy-back’ ride from another lysosomal protein to reach lysosomes in microglia. Nevertheless, our data support that PGRN takes multiple routes to the lysosome and each cell type might utilize different pathways for PGRN lysosomal delivery.

In agreement with the finding that PGRN is processed to granulin peptides in the lysosome, defects in PGRN lysosomal trafficking are associated with a decrease in the ratios between granulin peptides and full-length PGRN. Although the PSAP traffics with PGRN to the lysosome, PSAP does not seem to be required for PGRN, processing *per se* since PGRN can still be processed to the granulin peptides in PSAP-deficient cells and the defects are correlated with the lysosomal trafficking defects. It is possible that PSAP might affect the dynamics of PGRN processing and future work is required to fully examine this possibility.

Our analyses of PGRN processing and PGRN serum levels are consistent with the notion that PGRN lysosomal trafficking defects cause concomitant decrease in the levels of granulin peptides and increase in serum PGRN levels. The ablation of sortilin in mice has been shown to result in a significant increase in serum PGRN levels^30^ and the increases in the sortilin levels due to the single nucleotide polymorphism (SNP) have been associated with decreased plasma levels of PGRN in humans.^48^ Thus, sortilin has become a hot target for FTLD-*GRN* therapeutics to boost circulating PGRN levels. Small molecules and anti-sortilin antibodies have been developed to inhibit PGRN–sortilin interaction and anti-sortilin antibodies are currently in clinical trials for FTLD-*GRN*^49–51^ (NCT04111666, NCT04374136, NCT03987295). Recent studies have shown that anti-sortilin antibodies not only block PGRN–sortilin interaction but also downregulate sortilin levels.^49^ However, given the critical role of PGRN and granulin peptides in the lysosome^10,22^ and the fact that *GRN* mutations in FTLD also cause haploinsufficiency of granulin peptides,^33^ restoration of both extracellular and lysosomal pools of PGRN in the brain of FTLD-*GRN* patients will be needed to have better therapeutic outcomes.

## Supplementary Material

fcab310_Supplementary_Data

## Abbreviations

FTLD =: frontotemporal lobar degeneration
GRN =: granulin
LRP1 =: low-density lipoprotein receptor-related protein 1
M6PR =: mannose 6-phosphate receptor
NCL =: neuronal ceroid lipofuscinosis
PGRN =: progranulin
PSAP =: prosaposin
Sort =: sortilin
