# Supplementary material for: Regulation of lysosomal trafficking of progranulin by sortilin and prosaposin
Source: Brain Commun. 2022 Jan 4;4(1):fcab310. doi: 10.1093/braincomms/fcab310 (PMC8833632; doi:10.1093/braincomms/fcab310)
Supplement: fcab310_Supplementary_Data [file fcab310_supplementary_data.zip › Original Submission.pdf]

OXFORD
UNIVERSITY PRESS

Brain Communications

Regulation of progranulin lysosome trafficking by sortilin and prosaposin

Journal:	*Brain Communications*
Manuscript ID	BRAINCOM-2021-292
Manuscript Type:	Original Article
Date Submitted by the Author:	20-Aug-2021
Complete List of Authors:	Du, Huan; Cornell University, Weill Institute for Cell and Molecular Biology<br>Zhou, Xiaolai; Sun Yat-Sen University, State Key Laboratory of Ophthalmology<br>Feng, Tuancheng; Cornell University,<br>Hu, Fenghua; Cornell University, Weill Institute for Cell and Molecular Biology; Cornell University
Keywords:	progranulin, sortilin, prosaposin, FTLD, lysosome, granulin

SCHOLARONE™
Manuscripts

Regulation of progranulin lysosome trafficking by sortilin and prosaposin

Huan Du[1], Xiaolai Zhou[1,2], Tuancheng Feng[1] and Fenghua Hu[1]

[1]Department of Molecular Biology and Genetics, Weill Institute for Cell and Molecular Biology, Cornell University, Ithaca, NY 14853, USA

[2]State Key Laboratory of Ophthalmology, Zhongshan Ophthalmic Center, Sun Yat-sen University, Guangzhou, China

*Running Title: Lysosomal trafficking of PGRN

[#]To whom correspondence should be addressed: Fenghua Hu, 345 Weill Hall, Ithaca, NY 14853, TEL: 607-2550667, FAX: 607-2555961, Email: fh87@cornell.edu

For Review Only

Abstract

Haploinsufficiency of the progranulin (PGRN) protein is a leading cause of frontotemporal lobar degeneration (FTLD). Accumulating evidence support a crucial role of PGRN in the lysosome. PGRN is comprised of 7.5 granulin modules and is known to traffic to lysosomes via direct interactions with prosaposin (PSAP) or sortilin. Within the lysosome, PGRN gets processed into granulin peptides. Here we report that sortilin and PSAP independently regulate PGRN lysosome trafficking in vivo. Deletion of PSAP or sortilin alone has a mild effect but deficiency of both leads to a drastic decrease in the ratio of granulin peptides versus full length PGRN. A concomitant increase in the levels of secreted PGRN in the serum was observed. Interestingly, while deletion of PSAP and sortilin totally abolishes PGRN lysosomal localization in neurons, it has a limited effect on PGRN lysosomal trafficking in microglia, suggesting the existence of a novel sortilin and PSAP independent pathway for PGRN trafficking. In summary, our studies shed light on the regulation of PGRN lysosome trafficking and processing in vivo.

For Review Only

Introduction

Frontotemporal lobar degeneration (FTLD) is a devastating neurodegenerative disease that affects approximately 250,000 people in the United States[1,2]. Heterozygous mutations in the *granulin (GRN)* gene, resulting in progranulin (PGRN) haploinsufficiency, are one of the major causes of FTLD with TDP-43 and ubiquitin positive inclusions (FTLD-TDP)[3-5]. PGRN is an evolutionarily conserved, secreted glycoprotein of 88 kDa, comprised of 7.5 granulin modules [6 7-10]. The neurotrophic and anti-inflammatory functions of PGRN and granulin peptides are thought to prevent neurodegeneration in the aging brain[9,11-21]. However, multiple recent studies have suggested a critical role of PGRN in the lysosome [22]. First, homozygous PGRN mutant human patients exhibit neuronal ceroid lipofuscinosis (NCL), a lysosomal storage disorder [23,24]. PGRN knockout mice also accumulate the lysosomal byproduct, lipofuscin[17]. More importantly, NCL-related phenotypes are reported in FTLD patients with *GRN* mutation [25-27], supporting the theory that lysosomal dysfunction might serve as a common pathogenetic mechanism of these two diseases. Secondly, PGRN is transcriptionally co-regulated with a number of essential lysosomal genes by the transcriptional factor TFEB, a master regulator of lysosomal biogenesis [28,29]. Finally, PGRN has been shown to be a lysosome resident protein and there are two independent pathways for PGRN lysosome trafficking. Sortilin, a trafficking receptor of the vacuolar protein sorting 10 (VPS10) family, was shown to interact with PGRN and traffic PGRN to the lysosome [30]. In addition, PGRN interacts with another lysosomal protein prosaposin (PSAP) to get a "piggy-back" ride to the lysosome through PSAP receptors mannose 6-phosphate receptor (M6PR) and low density lipoprotein receptor-related protein 1 (LRP1) in a sortilin independent manner [31]. In the lysosome, PSAP is processed to saposin peptides, which regulate enzymes involved in glycosphingolipid degradation [32]. Interestingly, recently several studies have shown thatPGRN is processed in a similar manner to individual granulin peptides in the lysosome and several proteases, including cathepsin B, L and D have been implicated in the process [33-35]. The granulin peptides have been have been shown to regulate the activities of several lysosome enzymes, including cathepsin D [27,36-38] and glucocerebrosidase [39-41]. Furthermore, heterozygous mutations in the *granulin (GRN)* gene not only leads to the haplo-insufficiency of full length PGRN, but also decreased levels of granulin peptides (Holler et al., 2017).

Anti-sortilin antibodies which block PGRN-sortilin interaction are currently in clinical trials attempting to boost circulating PGRN levels to treat FTLD patients with *GRN* mutations (NCT04111666, NCT04374136, NCT03987295). Thus it is important to examine the effect of sortilin ablation on PGRN trafficking and processing *in vivo*. In this study, we generated mice deficient in both PSAP and sortilin and analyzed PGRN lysosome localization, the levels of granulin peptides as well as PGRN levels in serum in mice deficient in sortilin, PSAP or both. Our results further confirms that PSAP and sortilin are two independent lysosome trafficking pathways for PGRN and supports the existence of additional lysosomal trafficking pathway of PGRN in microglia.

Experimental procedures

For Review Only

Primary Antibodies and Reagents

The following antibodies were used in this study: Sheep anti-PGRN and granulin peptides (R&D Systems, AF2557) (1:750 for Western blot, 1:150 for Immunofluorescence staining), mouse anti-GAPDH (Proteintech Group, 60004-1-Ig), rat anti-mouse LAMP1 (BD Biosciences, 553792), rabbit anti IBA-1 (Wako, 01919741), goat anti-sortilin (Novus Biologicals, NB100-1028), and rabbit anti-PSAP antibodies [31] .

The following reagents were also used in the study: Dulbecco's modified Eagle's medium (DMEM)(Cellgro, 10-017-CV), 0.25% Trypsin (Corning, 25-053-CI), Odyssey blocking buffer (LI-COR Biosciences, 927-40000), TrueBlack Lipofuscin Autofluorescence Quencher (Biotium, 23007), protease inhibitor (Roche, 05056489001), Pierce BCA Protein Assay Kit (Thermo scientific, 23225), O.C.T compound (Electron Microscopy Sciences, 62550-01) and mouse PGRN ELISA Kit (BioLegend, 430901).

For Review Only

Mouse Strains

C57/BL6 and *Grn*$^{-/-}$ [42] mice were obtained from the Jackson Laboratory. Sortilin knockout mice (Nykjaer et al., 2004) were a gift from S. Strittmatter (Yale University, New Haven, CT) and A. Nykjaer (Aarhus University, Aarhus, Denmark). PSAP knockout mice were previously described (Fujita et al., 1996). *Psap*$^{+/-}$ and *Sort*$^{-/-}$ mice was mated to generate *Psap*$^{+/-}$ *Sort*$^{+/-}$ *mice*; *Psap*$^{+/-}$ *Sort*$^{+/-}$ mice were bred with each other to generate *Psap*$^{+/-}$ *Sort*$^{-/-}$ mice, which were mated to give *Psap*$^{-/-}$ *Sort*$^{-/-}$ mice. Sortilin genotyping was performed using the following primers: Forward WT, 5'-AAACAATCCTTCCATACCCAC-3'; Reverse WT, 5'-CCCCTTGTATTTCCTGTGGAC-3'; Reverse sortilin knockout, 5'-GATTGGGAAGACAATAGCAGG-3' (850-bp WT, 600-bp *Sort*$^{-/-}$). PSAP genotyping was performed using the following primers 5'-TTCAGCAAGTTCCCAGCTTCGG-3'; 5'-GAGCCCAATTTTAGCAAGAGA-3' (312-bp WT, 1500-bp *Psap*$^{-/-}$*)*. The age of the mice used was described in the figure legend for each experiment. Both male and female mice were used and the gender of the mice in each experiment was matched in the same experiment. All the mice were housed in the Weill Hall animal facility at Cornell. All animal procedures have been approved by the Institutional Animal Care and Use Committee (IACUC) at Cornell.

Cell Culture and biochemical assays

BV2 cells were maintained in Dulbecco's Modified Eagle's Medium (Cellgro) supplemented with 10% fetal bovine serum (Sigma) in a humidified incubator at 37ºC with 5% CO_2. To generate CRISPR constructs against mouse PSAP, two oligonucleotides with the sequences 5′-CACCGAAGAGGGCGAGGGCGTACA-3′ and 5′-AAACTGTACGCCCTCGCCCTCTTC-3′ were annealed and ligated to pLenti-CRISPRv2 (Addgene). Lentiviruses were generated by transfecting HEK293T cells with pLenti-CRISPRv2 constructs together with pMD2.G and psPAX2 plasmids. For CRISPR-mediated genome editing, BV2 cells were infected with lentiviruses

containing pLenti-CRISPRv2 harboring guide RNA sequences targeted to mouse PSAP. Cells were selected with puromycin (2µg/ml) 7 days after infection and the knockout is confirmed by western blot and immunostaining.

Western blot analysis

Mice were perfused with PBS and tissues were dissected and snap-frozen with liquid nitrogen and kept at -80°C. On the day of the experiment, frozen tissues were thawed and homogenized on ice with bead homogenizer (Moni International) in ice cold RIPA buffer (150 mM NaCl, 50 mM Tris-HCl [pH 8.0], 1% Triton X-100, 0.5% sodium deoxycholate, 0.1% SDS) with 1 mM PMSF, and 1x protease inhibitors (Roche). After centrifugation at 14,000 × g for 15 minutes at 4℃, supernatants were collected. Protein concentrations were determined via BCA assay, then standardized. Samples were separated by 4-12% Bis-Tris PAGE (Invitrogen) and transferred to 0.2µm nitrocellulose. Membranes were blocked with LiCor Odyssey blocking buffer for 2 h at room temperature followed by incubation with primary antibody overnight at 4°C with gentle rocking. Membranes were then washed with Tris-buffered saline with 0.1% Tween-20 (TBST) three times, 10 minutes each, and incubated with fluorescently tagged secondary antibodies (LI-COR Biosciences) for one hour at room temperature, followed by three washes. Membranes were scanned using an Odyssey Infrared Imaging System (LI-COR Biosciences). Densitometry was performed using Image Studio (LI-COR Biosciences) and Image J.

Immunofluorescence staining, image acquisition and analysis

For brain section staining, mice were perfused with cold PBS and tissues were post-fixed with 4% paraformaldehyde. After dehydration in 30% sucrose buffer, tissues were embedded in O.C.T compound (Electron Microscopy Sciences). 20-µm-thick brain sections were cut with cryotome. Tissue sections were blocked and permeabilized with 0.1% saponin in Odyssey blocking buffer before incubating with primary antibodies overnight at 4℃. The next day, sections were washed 3x with cold PBS followed by incubation with secondary fluorescent antibodies and Hoechst at room temperature for one hour. The slides were then mounted using mounting medium (Vector laboratories). To block the autofluorescence in aged mice, brain sections from 12-month-old mice were incubated with 1× TrueBlack Lipofuscin Autofluorescence Quencher (Biotium) in 70% ethanol for 30 seconds at room temperature after the staining process. Images were acquired on a CSU-X spinning disc confocal microscope (Intelligent Imaging Innovations) with an HQ2 CCD camera (Photometrics) using 100x objectives, ten to twelve different random images were captured. Lower magnification images were captured by 20x objectives on a Leica DMi8 inverted microscope, three to five images were captured from each sample. Data from ≥3 brains in each genotype were used for quantitative analysis.

For the quantitative analysis of neuronal PGRN levels in the brain sections, first neurons were selected based on the size of nuclei and PGRN expression, then the fluorescence intensity and area were measured directly using

For Review Only

ImageJ after a threshold application. To quantify microglia PGRN levels in the brain sections, microglia was selected based on microglia maker IBA1 staining and the images were analyzed using the same procedure in ImageJ. Total fluorescence signals were quantified. To quantify the degree of colocalization between PGRN and the lysosomal marker LAMP1, the JACoP plugin was used to generate Manders' overlap coefficients [43]. Three brain sections per mouse, separated by 100 μm were used for quantification. The mean from the three sections was used to be representative of each mouse. Data were normalized to age-matched controls.

RT-qPCR

Mouse cortical tissue was dissected and frozen in liquid nitrogen. Total RNAs were extracted using TRIzol (Invitrogen) and purified with Quick RNA MiniPrep Kit (Zymo Research). One microgram of total RNA was reverse transcribed to cDNA using poly (T) primer and SuperScript III Reverse Transcriptase (Invitrogen). Quantitative PCR was performed on a Light-Cyler 480 (Roch Applied Science), and the transcripts levels were measured using efficiency-adjusted ΔΔ−CT. PGRN transcript was normalized to β-actin and TBP. The mouse PGRN primer pair sequences were 5′-ATGTGGGTCCTGATGAGCTG-3′ and 5′-GCTCGTTATTCTAGGCCATGTG-3′. Mouse β-actin primers were 5′-ACGAGGCCCAGAGCAAGAG-3′ and 5′-TCTCCAAGTCGTCCCAGTTG-3′. Mouse TBP primers were 5′-CCCCACAACTCTTCCATTCT-3′ and 5′-GCAGGAGTGATAGGGGTCAT-3′.

Enzyme-linked immunosorbent assay

Mouse serum were collected and analyzed using mouse PGRN ELISA kit (R&D Systems, Catalog No. DY2557) according to the manufacturer's instructions as previously described [31].

ew Only

Statistical analysis

All statistical analyses were performed using GraphPad Prism 8. All data are presented as mean ± SEM. Statistical significance was assessed by unpaired Student's *t* test (for two groups comparison) or one-way ANOVA tests with Bonferroni's multiple comparisons (for multiple comparisons). P values less than or equal to 0.05 were considered statistically significant. $*p < 0.05$; $**p < 0.01$.

Results

PGRN trafficking and processing in sortilin deficient mice

Previously, we have shown that ablation of sortilin in mice leads to an increase of PGRN levels in the brain lysates and in the serum [30]. To examine the role of sortilin in PGRN trafficking more thoroughly, we stained brain

sections from age matched WT and *Sort*$^{-/-}$ mice with antibodies against PGRN, the main lysosomal membrane protein, LAMP1 and microglia maker IBA1. Consistent with our previous findings, we found that neuronal signals of PGRN and more specifically, lysosomal pool of PGRN in neurons are significantly affected by sortilin ablation in both 3-month-old and 12-month-old mice (Fig. 1a,b, Fig. S1). However, microglial PGRN remains localized in the lysosome compartment in *Sort*$^{-/-}$ brain sections and microglia PGRN levels are not altered (Fig. 1a,c), which is consistent with low expression levels of sortilin in microglia compared to neurons [30].

Next we examined the levels of granulin peptides and PGRN in the cortical lysates from 3-month-old WT and *Sort*$^{-/-}$ mice by western blot analyses, using polyclonal antibodies which recognize both full length PGRN and granulin peptides [44]. Concomitant with an increase in the levels of full length PGRN in *Sort*$^{-/-}$ mice [30], which is likely to be secreted, a significant reduction in the levels of granulin peptides were observed in the cortical lysates from *Sort*$^{-/-}$ mice, which results in a significance decrease in the ratio between granulin peptides and full length PGRN (Fig. 2). Thus lysosomal trafficking defects caused by sortilin deletion is associated with a decrease in PGRN processing.

PGRN trafficking and processing in PSAP deficient mice

Previously we have shown that PSAP interacts with PGRN and mediates sortilin independent lysosomal trafficking of PGRN [31]. To further determine the role of PSAP in PGRN processing *in vivo*, we examined the levels of both granulin peptides and PGRN in the cortical lysates from postnatal day 21 (P21) WT and *Psap*$^{-/-}$ mice by western blot analyses, since *Psap*$^{-/-}$ mice die around weaning age [45]. Ablation of PSAP leads to severe lysosomal abnormalities [31,45]and thus a significant increase in PGRN mRNA levels (Fig. S2) and levels of both full length PGRN and granulin peptides in the brain (Fig. 3). However, a significant decrease in the ratio between granulin peptides and full length PGRN was observed, which supports that PSAP plays a critical role in regulating PGRN lysosomal trafficking *in vivo*.

Ablation of PSAP and sortilin leads to defects in lysosomal trafficking and processing of PGRN

To further analyze the role of PSAP and sortilin in PGRN trafficking *in vivo*, we generated mice with both sortilin and PSAP ablated. Then we analyzed PGRN lysosomal localization in brain sections from *Psap*$^{-/-}$ *Sort*$^{-/-}$ mice. The PGRN signals retained in the lysosome compartment in *Sort*$^{-/-}$ neurons are completely lost in *Psap*$^{-/-}$ *Sort*$^{-/-}$ neurons (Fig. 4a, 4b), indicating that PSAP and sortilin are the only two pathways involved in PGRN lysosome trafficking in neurons. Significant co-localization between PGRN and lysosomal marker LAMP1 was observed in both *Psap*$^{-/-}$ and *Psap*$^{-/-}$ *Sort*$^{-/-}$ microglia (Fig. 4a), indicating that there exists additional PSAP and sortilin independent PGRN lysosome trafficking mechanism in microglia. Increased intensity of PGRN was observed in both *Psap*$^{-/-}$ and *Psap*$^{-/-}$ *Sort*$^{-/-}$ microglia due to transcriptional up-regulation of PGRN (Fig. S2). To further confirm this finding, we ablated PSAP in the microglial cell line BV2, which has very low levels of sortilin expression (Fig. S3), and

For Review Only

examined PGRN localization. PGRN remains localized in the lysosomal compartment in PSAP deficient BV2 cells, further supporting that PGRN lysosomal trafficking in microglia is sortilin and PSAP independent (Fig. 5a,5b).

Next we examined the levels of granulin peptides in the cortical lysates from 3-weeks-old WT, *Sort$^{-/-}$, Psap$^{-/-}$ Sort$^{-/-}$* mice as another readout of PGRN lysosomal trafficking. In the 3-week-old mice, ablation of sortilin results in a slight increase in the levels of both full length PGRN and granulin peptides. A slight decrease in the ratio between granulin peptides and PGRN was also observed (Fig. 6). Deficiency of both PSAP and sortilin results in an obvious increase in the levels of both PGRN and granulin peptides in the cortical lysates, with a significant decrease in the ratio between granulin peptides and full length PGRN (Fig. 6). However, there are still significant amount of granulin peptides present in *Psap$^{-/-}$ Sort$^{-/-}$* cortical lysates (Fig. 6a), consistent with our immunostaining results that PGRN lysosomal trafficking is affected in neurons but not microglia *Psap$^{-/-}$ Sort$^{-/-}$* mice (Fig. 4). Taken together, these data support that PSAP and sortilin serve as two independent lysosome trafficking pathways for PGRN *in vivo* and are the main two pathways to deliver PGRN to lysosomes in neurons, but there exist other sortilin and PSAP independent mechanisms to mediate PGRN lysosome trafficking in microglia, and possibly other cell types as well.

Disruption of lysosome trafficking also results in increased PGRN sorting to the secretory pathway and thus an increase in the serum levels of PGRN [30,31]. To further analyze the role of PSAP and sortilin in PGRN trafficking *in vivo*, we measured the levels of secreted PGRN in the serum. In the wild type (WT) mice, the concentration of PGRN in the serum is ~ 500 ng/ml, which is increased to ~3200ng/ml in *Sort$^{-/-}$* mice. Deletion of one copy of PSAP in *Sort$^{-/-}$* background (*Psap$^{+/-}$ Sort$^{-/-}$*) leads a further increase of PGRN levels to 4500ng/ml, indicating that PSAP haploinsufficiency exacerbates PGRN lysosome trafficking defects (Fig. 7a). Similarly, a further increase in PGRN serum levels was observed in *Psap$^{+/-}$ Sort$^{+/-}$* mice compared to *Psap$^{+/-}$* and *Sort$^{+/-}$* mice, which have already a significantly higher levels of serum PGRN compared to WT (Fig. 7b). In addition, ablation of both PSAP and sortilin leads to an increase of PGRN levels in the serum to ~17,000 ng/ml, which is much more than in *Psap$^{-/-}$* mice (~2500ng/ml) or *Sort$^{-/-}$* mice (~3200ng/ml) (Fig. 7c), further confirming that PSAP and sortilin are two independent lysosome trafficking pathways for PGRN *in vivo*.

Discussion

Previously, we have shown that PSAP and sortilin are two independent pathways mediating PGRN lysosomal trafficking [31,46]. This is further supported by our analyses of PGRN lysosomal localization and PGRN processing in *Psap$^{-/-}$, Sort$^{-/-}$* and *Psap$^{-/-}$ Sort$^{-/-}$* mice. While deletion of sortilin or PSAP alone results in partial defect of PGRN lysosomal trafficking, ablation of both PSAP and sortilin leads to nearly complete loss of lysosomal PGRN signals in neurons. However, PGRN remain localized in the lysosome in *Psap$^{-/-}$ Sort$^{-/-}$* microglia. In addition, PSAP deletion in BV2 microglial cell line, which expresses very low levels of sortilin (Fig. S2), does

For Review Only

not affect lysosomal trafficking of PGRN. All these data support that while PSAP and sortilin are the two main pathways mediating PGRN lysosomal trafficking in neurons, there exist additional PSAP and sortilin independent mechanisms in microglia and possibly other cell types to facilitate PGRN lysosomal delivery. It remains to be determined whether PGRN binds to a distinct lysosomal trafficking receptor or gets a "piggy-back" ride from another lysosomal protein to reach lysosomes in microglia. Nevertheless, our data support that PGRN takes multiple routes to the lysosome and each cell type might utilize different pathways for PGRN lysosomal delivery.

In agreement with the finding that PGRN is processed to granulin peptides in the lysosome, defects in PGRN lysosomal trafficking are associated with decreases in the ratios between granulin peptides and full length PGRN. Although PSAP traffics with PGRN to the lysosome, PSAP does not seem to be required for PGRN processing per se since PGRN can still be processed to granulin peptides in PSAP deficient cells and the defects are correlated with lysosomal trafficking defects. It is possible that PSAP might affect the dynamics of PGRN processing and future work is required to fully examine this possibility.

Our analyses of PGRN processing and PGRN serum levels are consistent with the notion that PGRN lysosomal trafficking defects cause concomitant decreases in the levels of granulin peptides and increases in serum PGRN levels. Ablation of sortilin in mice has been shown to result in a significant increase in serum PGRN levels [30] and the increases in sortilin levels due to single nucleotide polymorphism (SNP) have been associated with decreased plasma levels of PGRN in humans [47]. Thus, sortilin has become a hot target for FTLD-GRN therapeutics to boost circulating PGRN levels. Small molecules and anti-sortilin antibodies have been developed to inhibit PGRN-sortilin interaction and anti-sortilin antibodies are currently in clinical trials for FTLD-*GRN* [48-50] (NCT04111666, NCT04374136, NCT03987295). Recent studies have shown that anti-sortilin antibodies not only block PGRN-sortilin interaction but also downregulate sortilin levels [48] However, given the critical role of PGRN and granulin peptides in the lysosome [10,22] and the fact that *GRN* mutations in FTLD also cause haplo-insufficiency of granulin peptides (Holler et al., 2017), restoration of both extracellular and lysosomal pools of PGRN in the brain of FTLD-*GRN* patients will be needed to have better therapeutic outcomes.

Figure Legends

Figure 1: PGRN levels are decreased in neuron in Sortilin deficient mice. a Brain sections of 3-month-old WT and *Sort*$^{-/-}$ mice were stained with anti- PGRN, LAMP1 and IBA1antibodies. A representative neuron from the cortex was shown in inset (i) and a representative microglia was shown in inset (ii). Scale bar=10 µm. **b** Quantification of neuronal PGRN levels and overlap between PGRN and LAMP1 signals in neurons in a. Mean ± SEM; n=3, student's *t* test, **, p<0.01. **c** Quantification of microglia PGRN levels and overlap between PGRN and LAMP1 signals in microglia in a. Mean ± SEM; n=3, student's *t* test, ns, not significant.

Figure 2: PGRN processing in *Sort*$^{-/-}$ mice. a and b Western blot analysis of PGRN and granulin peptides in the cortical lysates from 3-month-old WT and *Sort*$^{-/-}$ mice. The levels of PGRN and granulin peptides (GRNs) were quantified and normalized to GAPDH. n=3, student's *t* test, *, p<0.05; **, p<0.01.

Figure 3: PGRN processing in PSAP deficient mice. a and b Western blot analysis of PGRN and granulin peptides in the cortical lysates from P21 WT and *Psap*$^{-/-}$ mice. The levels of PGRN and granulin peptides (GRNs) were quantified and normalized to GAPDH. n=3, student's *t* test, *, p<0.05, **, p<0.01.

Figure 4: PGRN levels are dramatically decreased in neuron in Sortilin and PSAP deficient mice. a Brain sections of P21 WT, *Psap*$^{-/-}$, *Sort*$^{-/-}$ and *Sort*$^{-/-}$ *Psap*$^{-/-}$ mice were stained with anti- PGRN, LAMP1 and IBA1antibodies. A representative neuron from the cortex was shown in inset (i) and representative microglia was shown in inset (ii). Scale bar=10 µm. **b** Quantification of neuronal PGRN levels in a. Mean ± SEM; n=3, one-way ANOVA, **, p<0.01.

Figure 5: PGRN trafficking are not changed in PSAP deficient BV2 cells. a Representative confocal images of BV2 cells stained with sheep anti-PGRN, rat anti-LAMP1 and rabbit anti-PSAP antibodies. Scale bar=10 µm. **b** Quantification of PGRN signals inside LAMP1-positive vesicles in a. Mean ± SEM; n=3, student's *t* test, ns, not significant.

Figure 6: PGRN processing in Sortilin and PSAP deficient mice. a and b Western blot analysis of PGRN and granulin peptides in the cortical lysates from P21 WT, *Sort*$^{-/-}$ and *Sort*$^{-/-}$ *Psap*$^{-/-}$ mice. The levels of PGRN and granulin peptides (GRNs) were quantified and normalized to GAPDH. n=3, one-way ANOVA, *, p<0.05; **, p<0.01.

Figure 7: Regulation of PGRN levels in serum by Sortilin and PSAP. a-c Serum PGRN levels in mice of different genotypes as indicated. Mean ± SEM; n=3, one-way ANOVA, *, p<0.05, **, p<0.01, ***, p<0.001.

For Review Only

Supplemental Material

Figure S1: PGRN levels are decreased in neuron in Sortilin deficient mice. a Brain sections of 12-month-old WT and *Sort*$^{-/-}$ mice were stained with anti- PGRN, LAMP1 and IBA1antibodies. A representative neuron was shown in inset i. Scale bar=10 µm. **b** Quantification of neuronal PGRN levels in a. Mean ± SEM; n=3, student's *t* test, **, p<0.01. **c** Quantification of neuronal PGRN puncta that are LAMP1-positive in a. Mean ± SEM; n=3, student's *t* test, **, p<0.01.

Figure S2: Quantitative PCR (qPCR) analysis of PGRN mRNA levels in the brain of P21 WT, *Psap*$^{-/-}$, *Sort*$^{-/-}$ and *Sort*$^{-/-}$ *Psap*$^{-/-}$ mice. Mean ± SEM; n=3, one-way ANOVA, *, p<0.05; **, p<0.01.

Figure S3: **Analysis of sortilin levels in N2A and BV2 cells.** Immunoblot for sortilin with lysates prepared from N2A and BV2 cells. GAPDH was used as a loading control.

Declarations

Abbreviations:

FTLD: frontotemporal lobar degeneration; NCL: neuronal ceroid lipofuscinosis; PGRN: progranulin; PSAP: prosaposin

Ethical Approval and Consent to Participate

All applicable international, national, and/or institutional guidelines for the care and use of animals were followed. The work under animal protocol 2017-0056 is approved by the Institutional Animal Care and Use Committee at Cornell University.

Consent for publication

All authors have given consent for publication.

Availability of data and material

All data have been included in the manuscript. Additional data are available upon request.

Competing interests

The authors declare that they have no competing interests.

Funding

This work is supported by NINDS/NIA (R01NS088448 & R01NS095954) and the Bluefield project to cure frontotemporal dementia to F.H.

Authors' contributions

H.D. and X. Z. performed all the experiments and analyzed the data. T.F. helped with mouse breeding and tissue collection. F.H. supervised the project and wrote the manuscript with H.D. X.Z. and T. F. edited the manuscript. All authors read and approved the final manuscript.

Acknowledgements

We thank Mrs. Xiaochun Wu for technical assistance.

For Review Only

REFERENCES

1 Neary, D., Snowden, J. & Mann, D. Frontotemporal dementia. *Lancet Neurol* **4**, 771-780, (2005).

2 Ratnavalli, E., Brayne, C., Dawson, K. & Hodges, J. R. The prevalence of frontotemporal dementia. *Neurology* **58**, 1615-1621, (2002).

3 Baker, M. *et al.* Mutations in progranulin cause tau-negative frontotemporal dementia linked to chromosome 17. *Nature* **442**, 916-919, (2006).

4 Cruts, M. *et al.* Null mutations in progranulin cause ubiquitin-positive frontotemporal dementia linked to chromosome 17q21. *Nature* **442**, 920-924, (2006).

5 Gass, J. *et al.* Mutations in progranulin are a major cause of ubiquitin-positive frontotemporal lobar degeneration. *Hum Mol Genet* **15**, 2988-3001, (2006).

6 Nicholson, A. M., Gass, J., Petrucelli, L. & Rademakers, R. Progranulin axis and recent developments in frontotemporal lobar degeneration. *Alzheimers Res Ther* **4**, 4, (2012).

7 Bateman, A. & Bennett, H. P. Granulins: the structure and function of an emerging family of growth factors. *J Endocrinol* **158**, 145-151, (1998).

8 Zhu, J. *et al.* Conversion of proepithelin to epithelins: roles of SLPI and elastase in host defense and wound repair. *Cell* **111**, 867-878, (2002).

9 Van Damme, P. *et al.* Progranulin functions as a neurotrophic factor to regulate neurite outgrowth and enhance neuronal survival. *J Cell Biol* **181**, 37-41, (2008).

10 Kao, A. W., McKay, A., Singh, P. P., Brunet, A. & Huang, E. J. Progranulin, lysosomal regulation and neurodegenerative disease. *Nat Rev Neurosci* **18**, 325-333, (2017).

11 Kleinberger, G. *et al.* Increased caspase activation and decreased TDP-43 solubility in progranulin knockout cortical cultures. *J Neurochem* **115**, 735-747, (2010).

12 Ryan, C. L. *et al.* Progranulin is expressed within motor neurons and promotes neuronal cell survival. *BMC Neurosci* **10**, 130, (2009).

13 Pereson, S. *et al.* Progranulin expression correlates with dense-core amyloid plaque burden in Alzheimer disease mouse models. *J Pathol* **219**, 173-181, (2009).

14 Gliebus, G., Rosso, A. & Lippa, C. F. Progranulin and {beta}-Amyloid Distribution: A Case Report of the Brain From Preclinical PS-1 Mutation Carrier. *Am J Alzheimers Dis Other Demen* **24**, 456-460, (2009).

15 Chen-Plotkin, A. S. *et al.* Brain progranulin expression in GRN-associated frontotemporal lobar degeneration. *Acta Neuropathol*, (2009).

16 Moisse, K. *et al.* Divergent patterns of cytosolic TDP-43 and neuronal progranulin expression following axotomy: implications for TDP-43 in the physiological response to neuronal injury. *Brain Res* **1249**, 202-211, (2009).

17 Ahmed, Z. *et al.* Accelerated lipofuscinosis and ubiquitination in granulin knockout mice suggest a role for progranulin in successful aging. *Am J Pathol* **177**, 311-324, (2010).

18 Ahmed, Z., Mackenzie, I. R., Hutton, M. L. & Dickson, D. W. Progranulin in frontotemporal lobar degeneration and neuroinflammation. *J Neuroinflammation* **4**, 7, (2007).

19 Yin, F. *et al.* Exaggerated inflammation, impaired host defense, and neuropathology in progranulin-deficient mice. *J Exp Med* **207**, 117-128, (2009).

20 Laird, A. S. *et al.* Progranulin is neurotrophic in vivo and protects against a mutant TDP-43 induced axonopathy. *PLoS ONE* **5**, e13368, (2010).

21 Xu, J. *et al.* Extracellular progranulin protects cortical neurons from toxic insults by activating survival signaling. *Neurobiol Aging* **32**, 2326 e2325-2316, (2011).

22 Paushter, D. H., Du, H., Feng, T. & Hu, F. The lysosomal function of progranulin, a guardian against neurodegeneration. *Acta Neuropathol* **136**, 1-17, (2018).

For Review Only

23 Almeida, M. R. *et al.* Portuguese family with the co-occurrence of frontotemporal lobar degeneration and neuronal ceroid lipofuscinosis phenotypes due to progranulin gene mutation. *Neurobiol Aging* **41**, 200 e201-205, (2016).
24 Smith, K. R. *et al.* Strikingly different clinicopathological phenotypes determined by progranulin-mutation dosage. *Am J Hum Genet* **90**, 1102-1107, (2012).
25 Ward, M. E. *et al.* Individuals with progranulin haploinsufficiency exhibit features of neuronal ceroid lipofuscinosis. *Sci Transl Med* **9**, eaah5642, (2017).
26 Gotzl, J. K. *et al.* Common pathobiochemical hallmarks of progranulin-associated frontotemporal lobar degeneration and neuronal ceroid lipofuscinosis. *Acta Neuropathol* **127**, 845-860, (2014).
27 Valdez, C. *et al.* Progranulin-mediated deficiency of cathepsin D results in FTD and NCL-like phenotypes in neurons derived from FTD patients. *Hum Mol Genet* **26**, 4861-4872, (2017).
28 Belcastro, V. *et al.* Transcriptional gene network inference from a massive dataset elucidates transcriptome organization and gene function. *Nucleic Acids Res* **39**, 8677-8688, (2011).
29 Sardiello, M. *et al.* A gene network regulating lysosomal biogenesis and function. *Science* **325**, 473-477, (2009).
30 Hu, F. *et al.* Sortilin-mediated endocytosis determines levels of the frontotemporal dementia protein, progranulin. *Neuron* **68**, 654-667, (2010).
31 Zhou, X. *et al.* Prosaposin facilitates sortilin-independent lysosomal trafficking of progranulin. *Journal of Cell Biology* **210**, 991-1002, (2015).
32 O'Brien, J. S. & Kishimoto, Y. Saposin proteins: structure, function, and role in human lysosomal storage disorders. *FASEB J* **5**, 301-308, (1991).
33 Holler, C. J., Taylor, G., Deng, Q. & Kukar, T. Intracellular Proteolysis of Progranulin Generates Stable, Lysosomal Granulins that Are Haploinsufficient in Patients with Frontotemporal Dementia Caused by GRN Mutations. *eNeuro* **4**, (2017).
34 Lee, C. W. *et al.* The lysosomal protein cathepsin L is a progranulin protease. *Mol Neurodegener* **12**, 55, (2017).
35 Zhou, X. *et al.* Lysosomal processing of progranulin. *Mol Neurodegener* **12**, 62, (2017).
36 Zhou, X. *et al.* Regulation of cathepsin D activity by the FTLD protein progranulin. *Acta Neuropathol* **134**, 151-153, (2017).
37 Beel, S. *et al.* Progranulin functions as a cathepsin D chaperone to stimulate axonal outgrowth in vivo. *Hum Mol Genet* **26**, 2850-2863, (2017).
38 Butler, V. J. *et al.* Progranulin Stimulates the In Vitro Maturation of Pro-Cathepsin D at Acidic pH. *J Mol Biol* **431**, 1038-1047, (2019).
39 Arrant, A. E. *et al.* Impaired beta-glucocerebrosidase activity and processing in frontotemporal dementia due to progranulin mutations. *Acta Neuropathol Commun* **7**, 218, (2019).
40 Valdez, C., Ysselstein, D., Young, T. J., Zheng, J. & Krainc, D. Progranulin mutations result in impaired processing of prosaposin and reduced glucocerebrosidase activity. *Hum Mol Genet* **29**, 716-726, (2019).
41 Zhou, X. *et al.* Progranulin deficiency leads to reduced glucocerebrosidase activity. *PLoS One* **14**, e0212382, (2019).
42 Yin, F. *et al.* Exaggerated inflammation, impaired host defense, and neuropathology in progranulin-deficient mice. *J Exp Med* **207**, 117-128, (2010).
43 Dunn, K. W., Kamocka, M. M. & McDonald, J. H. A practical guide to evaluating colocalization in biological microscopy. *Am J Physiol Cell Physiol* **300**, C723-742, (2011).
44 Zhang, T. *et al.* Differential Regulation of Progranulin derived granulin peptides. *biorxiv*, (2021).
45 Fujita, N. *et al.* Targeted Disruption of the Mouse Sphingolipid Activator Protein Gene: A Complex Phenotype, Including Severe Leukodystrophy and Wide-Spread Storage of Multiple Sphingolipids. *Human Molecular Genetics* **5**, 711-725, (1996).

For Review Only

46 Zhou, X. *et al.* Impaired prosaposin lysosomal trafficking in frontotemporal lobar degeneration due to progranulin mutations. *Nature communications* **8**, 15277, (2017).
47 Carrasquillo, M. M. *et al.* Genome-wide screen identifies rs646776 near sortilin as a regulator of progranulin levels in human plasma. *Am J Hum Genet* **87**, 890-897, (2010).
48 Miyakawa, S. *et al.* Anti-sortilin1 Antibody Up-Regulates Progranulin via Sortilin1 Down-Regulation. *Front Neurosci* **14**, 586107, (2020).
49 Stachel, S. J. *et al.* Identification of potent inhibitors of the sortilin-progranulin interaction. *Bioorg Med Chem Lett* **30**, 127403, (2020).
50 Lee, W. C. *et al.* Targeted manipulation of the sortilin-progranulin axis rescues progranulin haploinsufficiency. *Hum Mol Genet* **23**, 1467-1478, (2014).

For Review Only

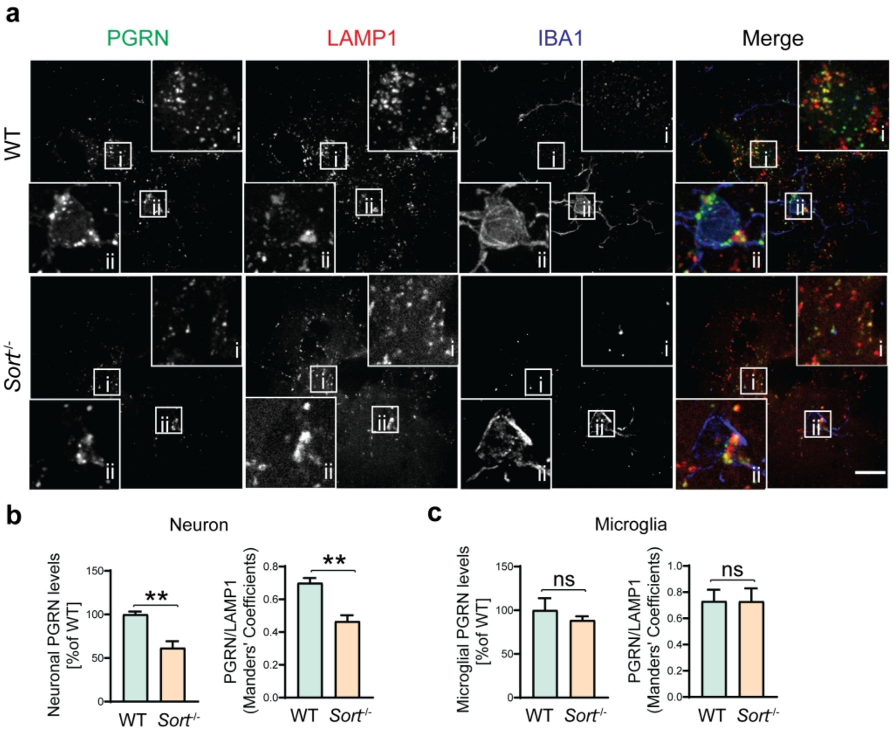

Fig 1

Figure 1: PGRN levels are decreased in neuron in Sortilin deficient mice. a Brain sections of 3-month-old WT and Sort-/- mice were stained with anti- PGRN, LAMP1 and IBA1antibodies. A representative neuron from the cortex was shown in inset (i) and a representative microglia was shown in inset (ii). Scale bar=10 μm. b Quantification of neuronal PGRN levels and overlap between PGRN and LAMP1 signals in neurons in a. Mean ± SEM; n=3, student's t test, **, p<0.01. c Quantification of microglia PGRN levels and overlap between PGRN and LAMP1 signals in microglia in a. Mean ± SEM; n=3, student's t test, ns, not significant.

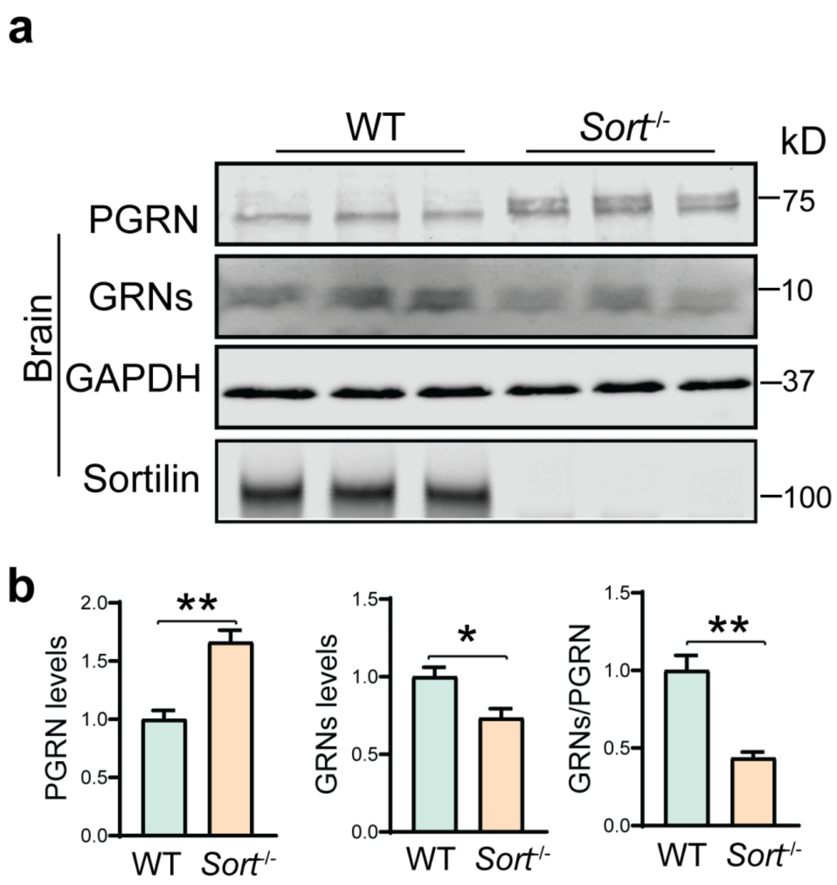

Fig 2

Figure 2: PGRN processing in Sort-/- mice. a and b Western blot analysis of PGRN and granulin peptides in the cortical lysates from 3-month-old WT and Sort-/- mice. The levels of PGRN and granulin peptides (GRNs) were quantified and normalized to GAPDH. n=3, student's t test, *, $p<0.05$; **, $p<0.01$.

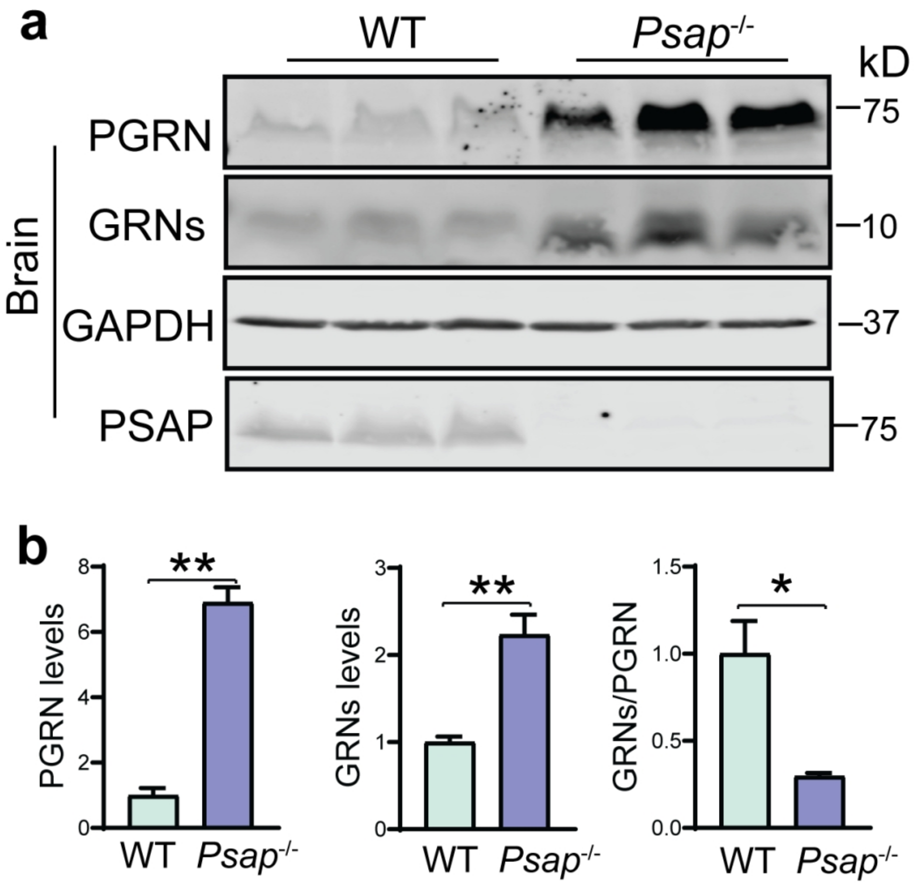

Fig 3

Figure 3: PGRN processing in PSAP deficient mice. a and b Western blot analysis of PGRN and granulin peptides in the cortical lysates from P21 WT and Psap-/- mice. The levels of PGRN and granulin peptides (GRNs) were quantified and normalized to GAPDH. n=3, student's t test, *, $p<0.05$, **, $p<0.01$.

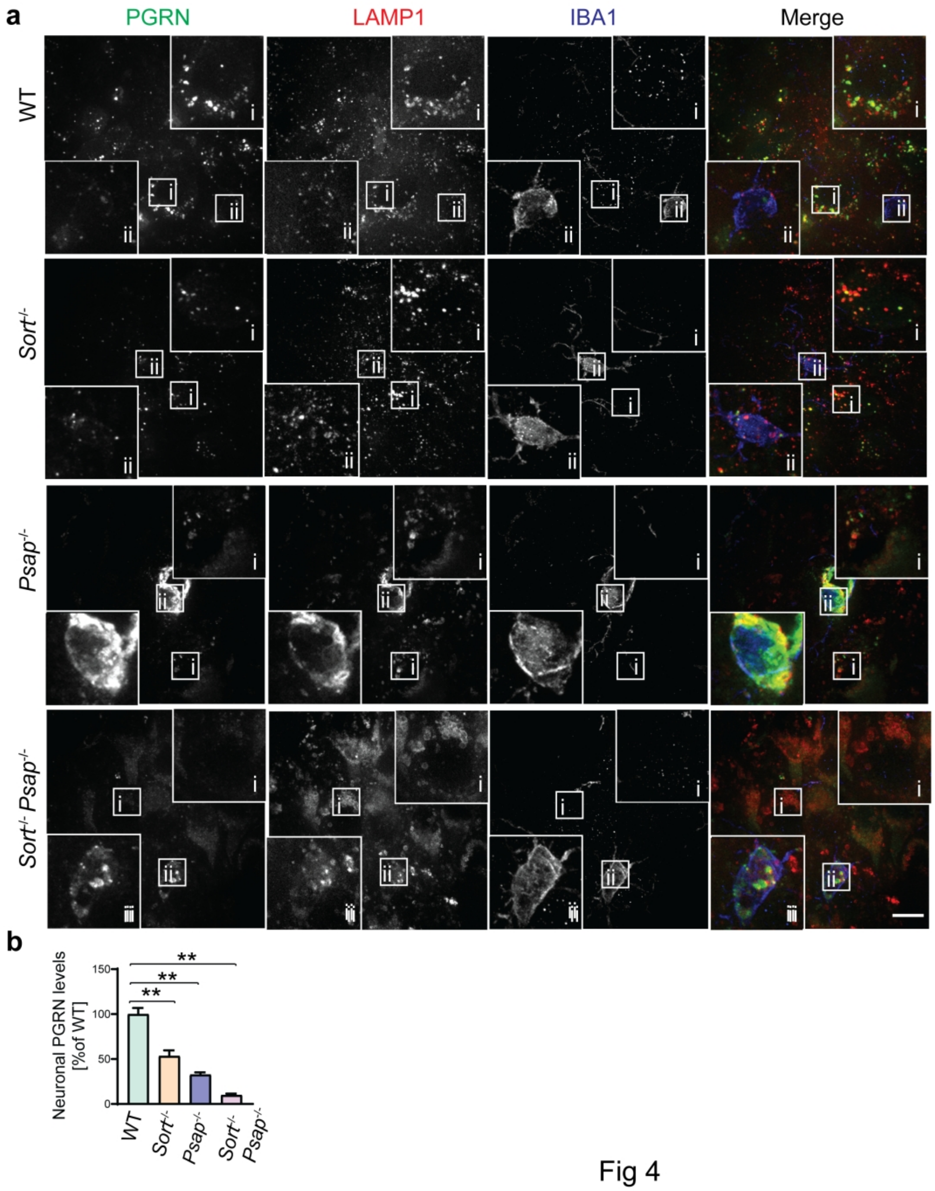

Fig 4

Figure 4: PGRN levels are dramatically decreased in neuron in Sortilin and PSAP deficient mice. a Brain sections of P21 WT, Psap-/-, Sort-/- and Sort-/- Psap-/- mice were stained with anti- PGRN, LAMP1 and IBA1antibodies. A representative neuron from the cortex was shown in inset (i) and representative microglia was shown in inset (ii). Scale bar=10 μm. b Quantification of neuronal PGRN levels in a. Mean ± SEM; n=3, one-way ANOVA, **, $p<0.01$.

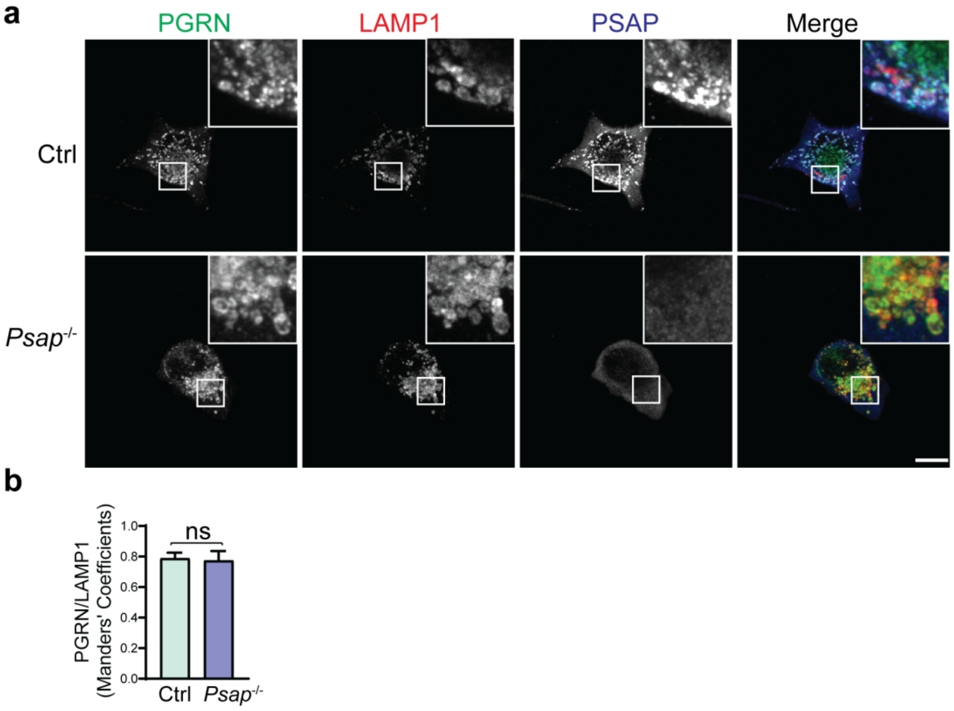

Fig 5

Figure 5: PGRN trafficking are not changed in PSAP deficient BV2 cells. a Representative confocal images of BV2 cells stained with sheep anti-PGRN, rat anti-LAMP1 and rabbit anti-PSAP antibodies. Scale bar=10 μm. b Quantification of PGRN signals inside LAMP1-positive vesicles in a. Mean ± SEM; n=3, student's t test, ns, not significant.

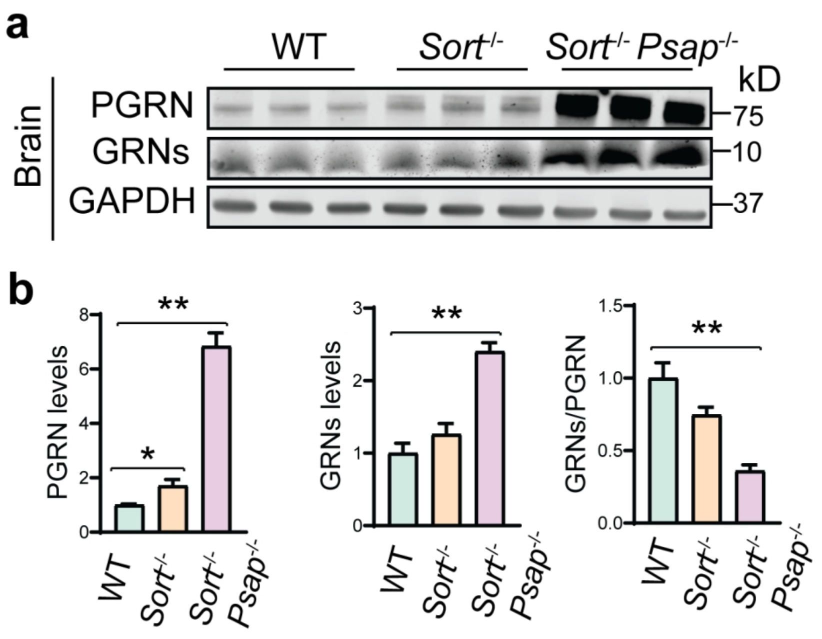

Fig 6

Figure 6: PGRN processing in Sortilin and PSAP deficient mice. a and b Western blot analysis of PGRN and granulin peptides in the cortical lysates from P21 WT, Sort-/- and Sort-/- Psap-/- mice. The levels of PGRN and granulin peptides (GRNs) were quantified and normalized to GAPDH. n=3, one-way ANOVA, *, $p<0.05$; **, $p<0.01$.

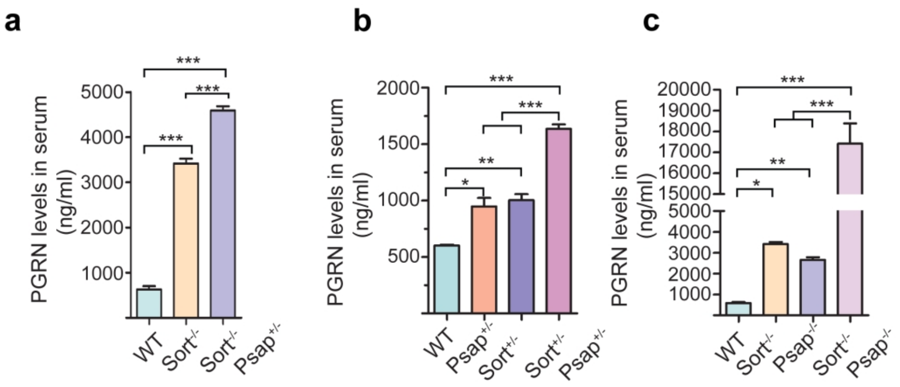

Fig 7

Figure 7: Regulation of serum PGRN levels by Sortilin and PSAP. a-c Serum PGRN levels in mice of different genotypes as indicated. Mean ± SEM; n=3, one-way ANOVA, *, p<0.05, **, p<0.01, ***, p<0.001.

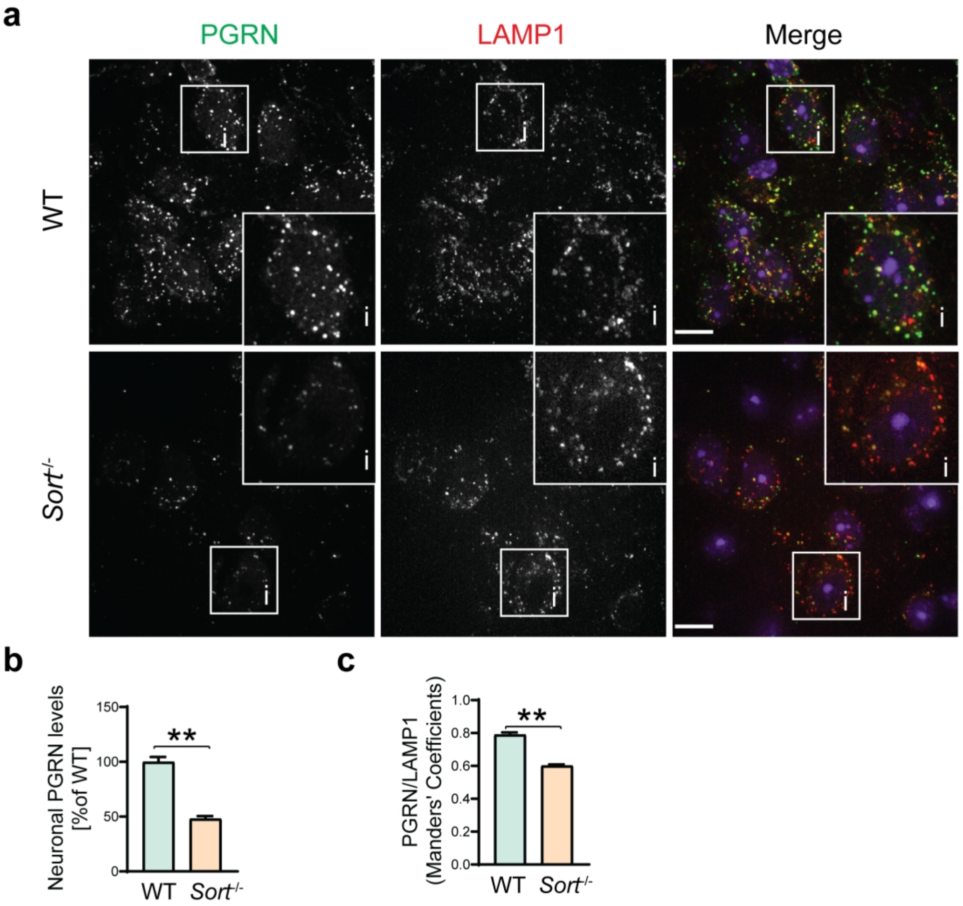

Fig S1

Figure S1: PGRN levels are decreased in neurons in Sortilin deficient mice. a Brain sections of 12-month-old WT and Sort-/- mice were stained with anti- PGRN, LAMP1 and IBA1antibodies. A representative neuron was shown in inset i. Scale bar=10 μm. b Quantification of neuronal PGRN levels in a. Mean ± SEM; n=3, student's t test, **, p<0.01. c Quantification of neuronal PGRN puncta that are LAMP1-positive in a. Mean ± SEM; n=3, student's t test, **, p<0.01.

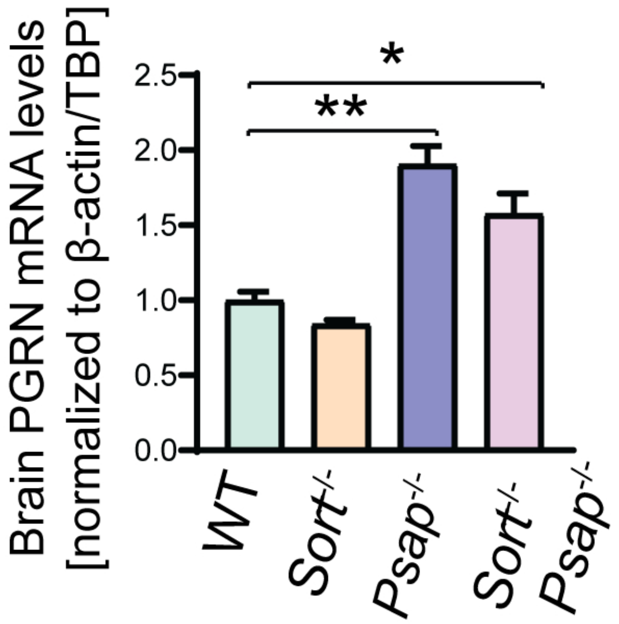

Fig S2

Figure S2: Quantitative PCR (qPCR) analysis of PGRN mRNA levels in the brain of P21 WT, Psap-/-, Sort-/- and Sort-/- Psap-/- mice. Mean ± SEM; n=3, one-way ANOVA, *, $p<0.05$; **, $p<0.01$.

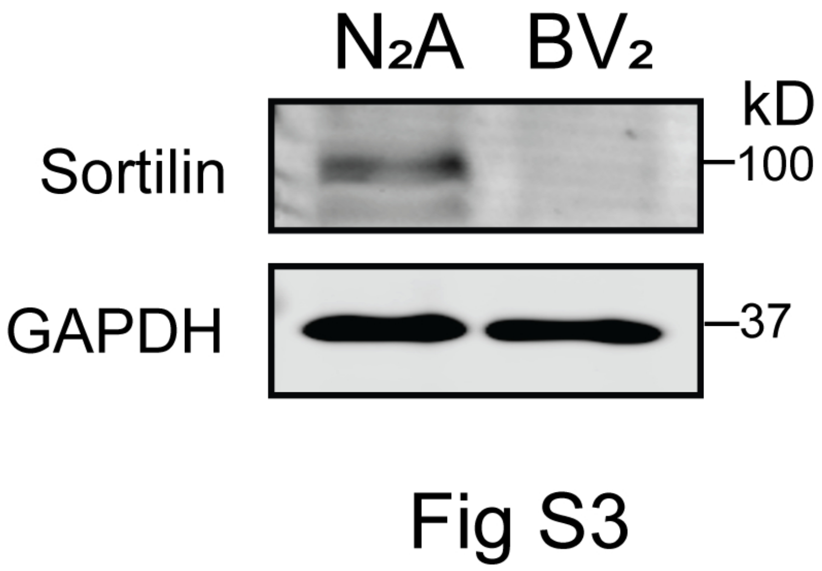

Figure S3: Analysis of sortilin levels in N2A and BV2 cells. Immunoblot for sortilin with lysates prepared from N2A and BV2 cells. GAPDH was used as a loading control.
