# Supplementary material for: Regulation of lysosomal trafficking of progranulin by sortilin and prosaposin
Source: Brain Commun. 2022 Jan 4;4(1):fcab310. doi: 10.1093/braincomms/fcab310 (PMC8833632; doi:10.1093/braincomms/fcab310)
Supplement: fcab310_Supplementary_Data [file fcab310_supplementary_data.zip › Supplementary material.pdf]

Supplementary Material

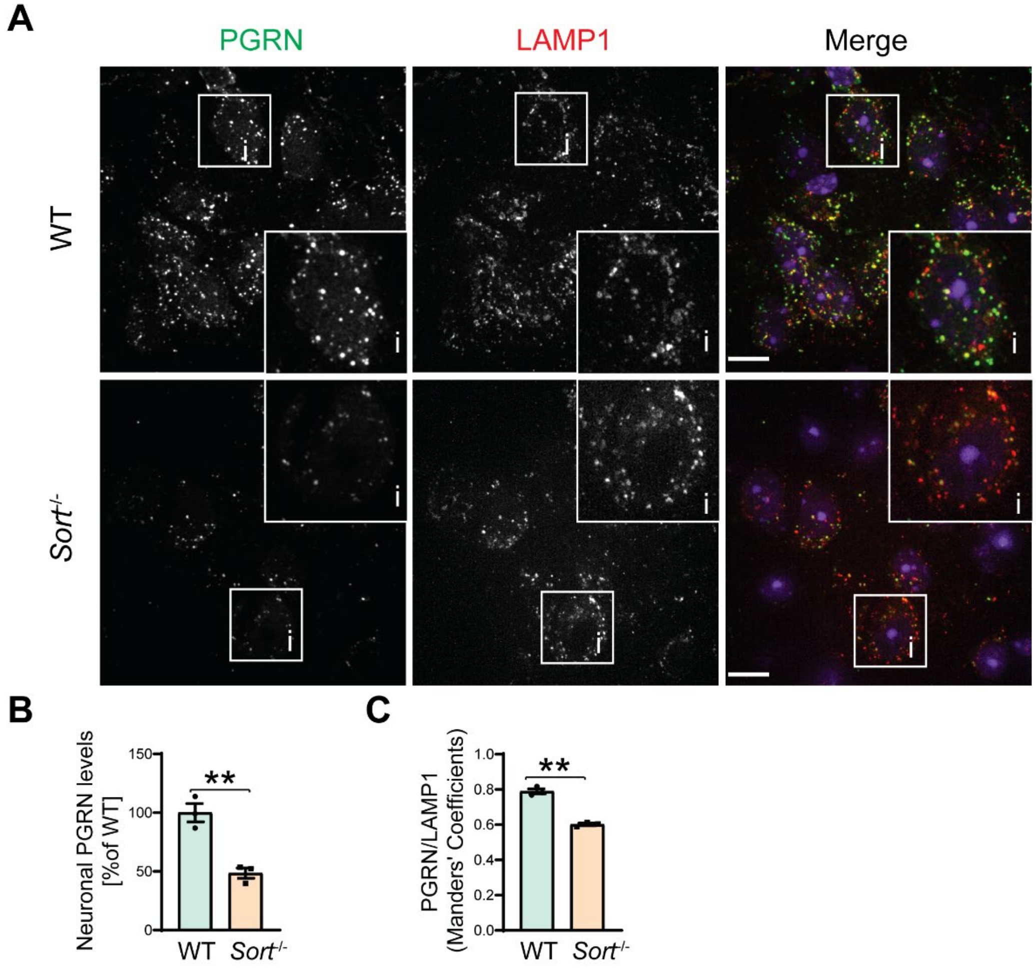

Figure S1: PGRN levels are decreased in neurons in Sortilin deficient mice. (A) Brain sections of 12-month-old WT and *Sort*$^{-/-}$ mice were stained with anti- PGRN, LAMP1 and IBA1antibodies. A representative neuron was shown in inset i. Scale bar=10 µm. **(B)** Quantification of neuronal PGRN levels in a. Mean ± SEM; n=3, student's *t* test, **, p<0.01. **(C)** Quantification of neuronal PGRN puncta that are LAMP1-positive in a. Mean ± SEM; n=3, student's *t* test, **, p<0.01.

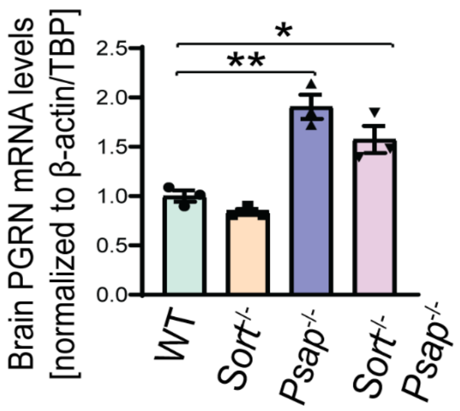

Figure S2: Quantitative PCR (qPCR) analysis of PGRN mRNA levels in the brain of P21 WT, *Psap*$^{-/-}$*, Sort*$^{-/-}$ and *Sort*$^{-/-}$ *Psap*$^{-/-}$ mice. Mean ± SEM; n=3, one-way ANOVA, *, p<0.05; **, p<0.01.

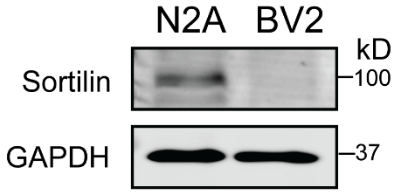

Figure S3: **Analysis of sortilin levels in N2A and BV2 cells.** Immunoblot for sortilin with lysates prepared from N2A and BV2 cells. GAPDH was used as a loading control.
