# Supplementary figures and images for: Regulation of lysosomal trafficking of progranulin by sortilin and prosaposin
Source: Brain Commun. 2022 Jan 4;4(1):fcab310. doi: 10.1093/braincomms/fcab310 (PMC8833632; doi:10.1093/braincomms/fcab310)

WT

*Sort<sup>-/-</sup>*

PGRN

GRNs

GAPDH

Sortilin

WT

*Psap<sup>-/-</sup>*

PGRN

GRNs

GAPDH

PSAP

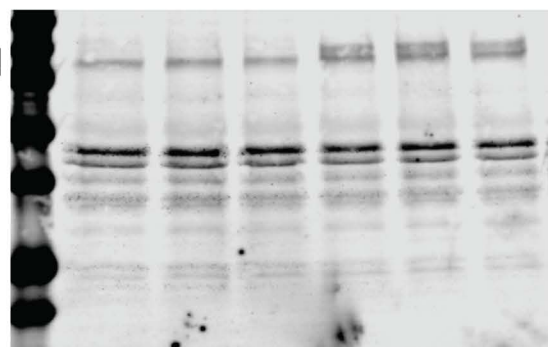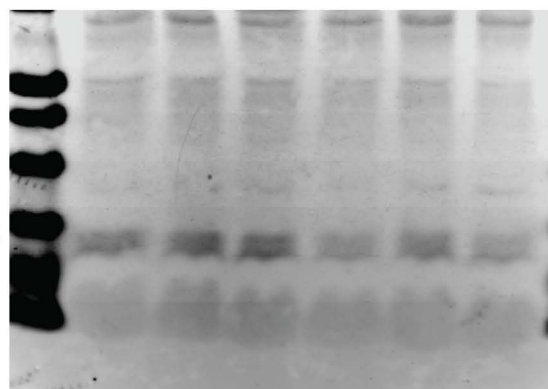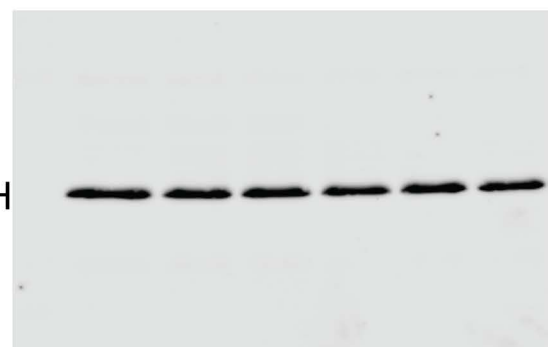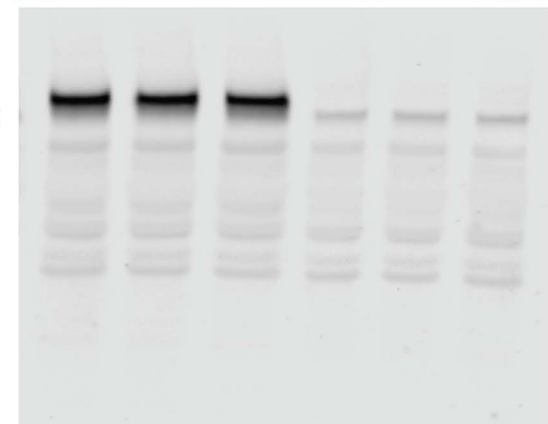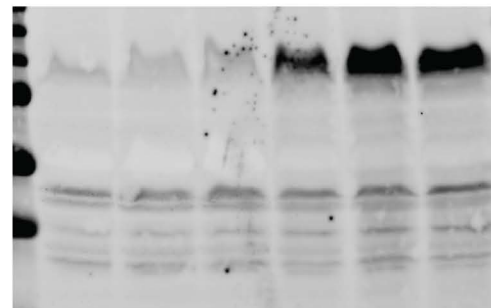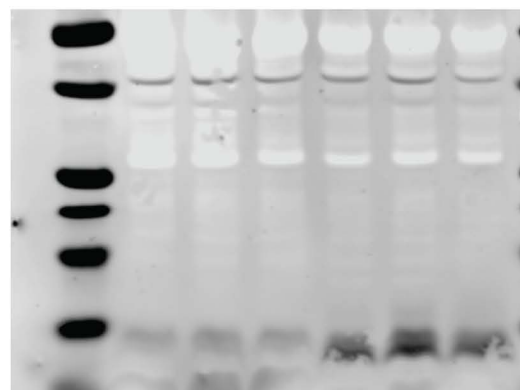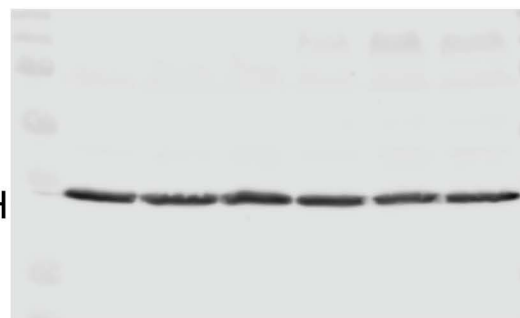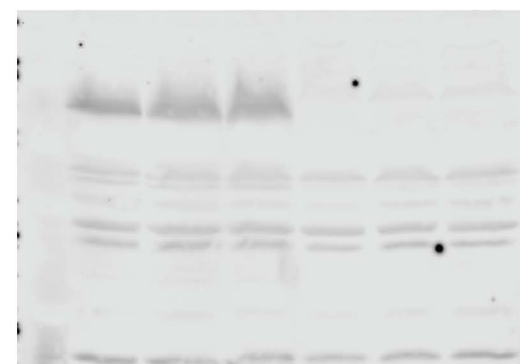

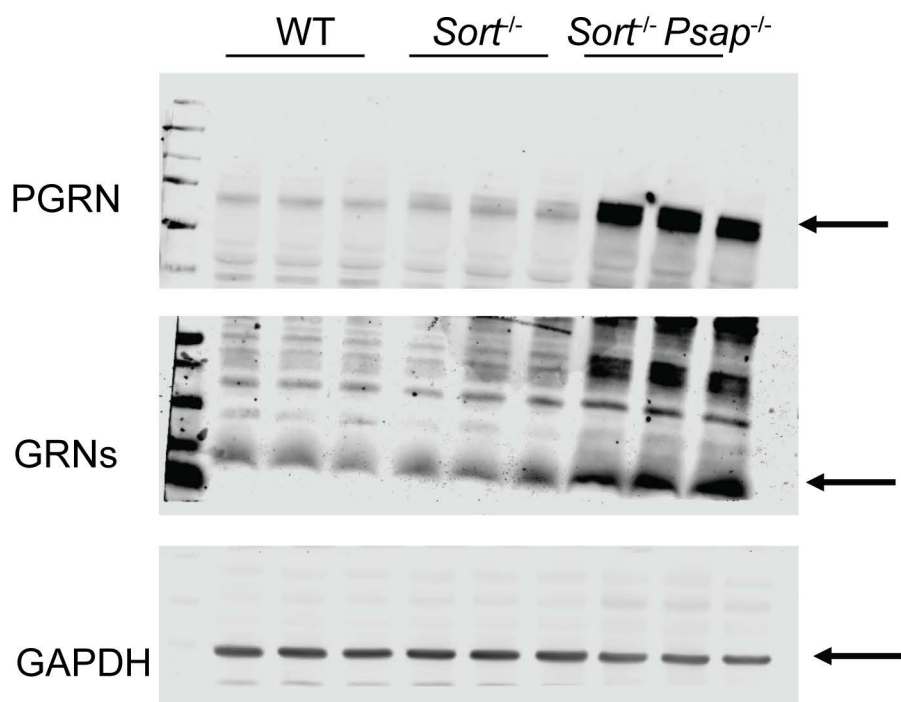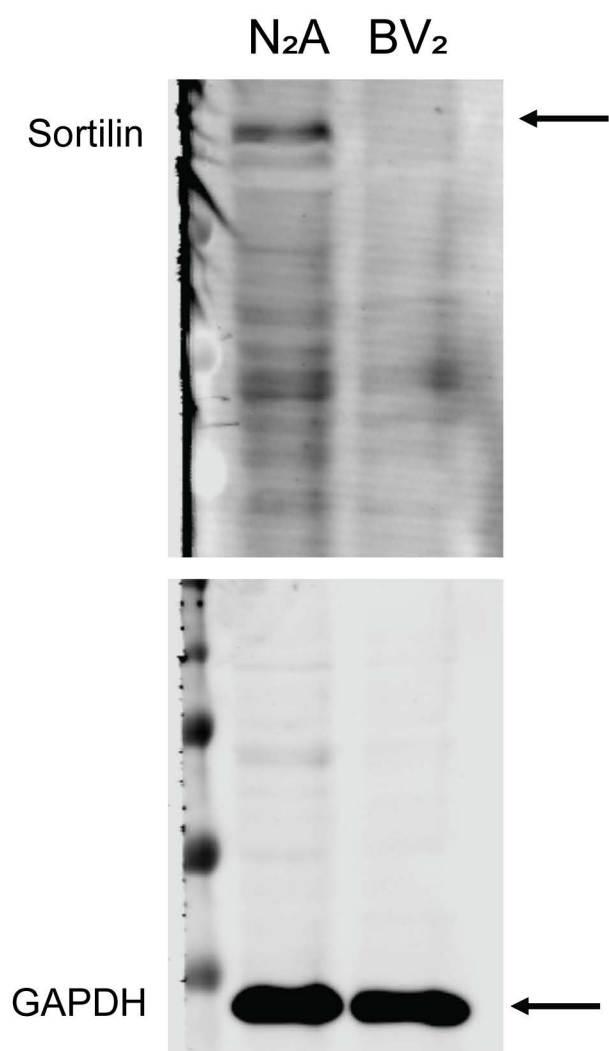

Supplement: fcab310_Supplementary_Data [file fcab310_supplementary_data.zip › Supplementary materal file 2.pdf]
