# Supplementary material for: Regulation of lysosomal trafficking of progranulin by sortilin and prosaposin
Source: Brain Commun. 2022 Jan 4;4(1):fcab310. doi: 10.1093/braincomms/fcab310 (PMC8833632; doi:10.1093/braincomms/fcab310)
Supplement: fcab310_Supplementary_Data [file Original Submission GÇô AuthorGÇÖs Response.pdf › Original Submission GÇô AuthorGÇÖs Response.pdf]

Original Submission – Author's Response

We thank the reviewers for careful review of our manuscript and for their constructive comments.

Reviewer: 1

Comments to the Author
Progranulin (PGRN) is implicated in the pathogenesis of a variety of diseases; however, it is most strongly tied to frontotemporal dementia. In this study, Du et al. demonstrated the role of sortilin and prosaposin (PSAP) in PGRN lysosome trafficking and processing in vivo. Authors generated mice deficient in PSAP and/or sortilin and analyzed PGRN lysosome localization, levels of granulin peptides and PGRN in serum.
Authors show that PSAP and sortilin are the only two pathways involved in PGRN lysosome trafficking in neurons, while PSAP and sortilin independent PGRN lysosome trafficking mechanism exists in microglia.

Overall, there are some minor concerns/suggestions about the experiments listed below.
1. Please mention the number of animals used in this study.
Reply: The number of animals used in the study is now described in the figure legends.

2. Biochemical analysis from neuronal and microglia enriched fractions from mice different ages should confirm the immunofluorescence results in figure 1 and 2 3. Microglia PGRN levels and PGRN/LAMP1 colocalization analysis should present in figure 4.
Reply: Thank you very much for the suggestions. We have quantified microglial PGRN levels and performed PGRN/LAMP1 colocalization analysis for the DKO mice (Fig. 4c). We agree with the reviewer that it will be nice to have biochemical analysis to confirm immunofluorescence results. However, isolating microglia from these mice is very challenging due to difficulties obtaining Psap-/- and DKO mice. Due to the early lethality of Psap-/- mice, we will need to breed Psap+/- mice with each other to get Psap-/- pups and breed Psap+/- Sort-/- mice with each other to get Psap-/- Sort-/- mice. Only 1/10 of the pups from these matings are Psap-/-.

4. It is good to have results from primary glial culture along with results in BV2 cells.
Reply: We agree. However, it is technical difficult to obtain enough Psap-/- pups to culture primary microglia due to the reason stated above.

5. Please check typo error in Introduction.
Reply: Thanks, we've corrected the typo.

Reviewer: 2

Comments to the Author
The authors present the potential mechanisms for lysosomal trafficking of PGRN via sortilin and PSAP binding. The work mainly expands on the previous findings by the same authors using mice models. They argue that in mice with PSAP and sortilin ablation, the levels of circulating PGRN is increased significantly along with increase in the levels of Grn peptides (cleavage products of PGRN), and significant decrease in the ratio of Grns to PGRN. Interestingly, the authors also argue that ablation of Sort and PSAP does not affect the lysosomal trafficking of PGRN in microglia and this trafficking is independent of Sort/PSAP pathway. The study is straightforward, simple, and well-designed. Following are some comments/questions that will further improve the study –
1. Can authors elaborate why the ratio of GRNS/PGRN is low in cortical lysates of PSAP-/- mice even as the levels of granulins are high? Same question in case of Sot-/- PSAP-/- mice.
Reply: We appreciate positive comments from the reviewer. PSAP deficiency leads to up-regulation of PGRN mRNA (Fig. S2), which results in an increase in the levels of both PGRN and granulin peptides (Fig. 3). However, because PSAP is critical for PGRN trafficking in neurons, PSAP deficiency leads to impaired lysosomal trafficking of PGRN and thus a decrease in PGRN processing rate, resulting in a decreased ratio of GRNs/PGRN (Fig. 3).

2. Since there is a decline in the Grns to PGRN ratio in PSAP -/- and sort-/- PSAP-/- mice, did the authors measure the ratio of Grns/PGRN in WT mice? Or is this ratio available in literature. If yes, can authors elaborate and compare with the Grns/PGRN ratio in PSAP and Sort-PSAP knockouts?
Reply: Yes, the ratio of Grns/PGRN in age matched WT mice is measured side by side with PSAP -/- and sort-/- PSAP-/- mice and our results have shown a decreased ratio of GRNs/PGRN in Psap-/- and Psap-/- Sort-/- mice compared to that in WT (Figs, 2,3 and 6).

3. Did the authors observe in phenotypic/behavioral changes in the mice with sortilin ablation? Since several clinical trials are looking at blocking sortilin receptors to increase circulation levels of PGRN in FTLD patients, it is important to see any behavioral changes in mice with sortilin ablation?
Reply: Thanks for the suggestion. We did not observe any obvious behavioral deficits in Sort-/- mice.

However, sortilin has been studied in the context of Pro-NgF signaling , neurotensin signaling and cholesterol metabolism among others. Phenotypic characterization of sort-/- mice has been reported by other groups (Such as Jansen et al, Nature Neuroscience volume 10, pages1449–1457 (2007); Devader et al, Front. Neurosci., 24 November 2016; Li et al, JBC 2017; Moreno et al, Front Pharmacol. 2018; 9: 863).

4. Can authors quantify the 'slight decrease' in the ratio of Grns/PGRN in Sort-/- mice (line 12, page 8)?
Reply: Yes, the ratio of Grns/PGRN in age matched WT and Sort-/- mice is quantified and shown in Fig.2.
