# Supplementary material for: Regulation of lysosomal trafficking of progranulin by sortilin and prosaposin
Source: Brain Commun. 2022 Jan 4;4(1):fcab310. doi: 10.1093/braincomms/fcab310 (PMC8833632; doi:10.1093/braincomms/fcab310)
Supplement: fcab310_Supplementary_Data [file Original Submission GÇô Decision Letter.pdf › Original Submission GÇô Decision Letter.pdf]

Original Submission – Decision Letter

01-Oct-2021

Dear Dr. Hu,

Thank you for your recent submission BRAINCOM-2021-292
entitled "Regulation of progranulin lysosome trafficking by sortilin and prosaposin" to Brain Communications.

Your manuscript has been reviewed, and while the reviewers had largely positive comments, they have some constructive critiques that I think you could address in a revision of the manuscript. Therefore, I invite you to respond to the reviewer(s)' comments and revise your manuscript. The reviewer(s)' comments are included at the bottom of this message.

In addition to the reviewers' comments, please also address our Scientific Editor's comments added below to comply with our requirements for presentation of scientific data at Brain Communications.

Please format your revised manuscript according to our Instructions to Authors https://academic.oup.com/braincomms/pages/General_Instructions – a summary of key points is attached to help with this.

To revise your manuscript, click the link below or log into https://mc.manuscriptcentral.com/braincom and enter your Author Centre, where you will find your manuscript title listed under "Manuscripts with Decisions." Under "Actions," click on "Create a Revision." Your manuscript number will be appended to denote a revision.

*** PLEASE NOTE: This is a two-step process. After clicking on the link, you will be directed to a webpage to confirm. ***

https://mc.manuscriptcentral.com/braincom?URL_MASK=156179bda8e0451dafb9ebfadfb19cba

Please upload a revised manuscript with the changes you have made shown either in coloured text or using "track changes".

When submitting your revised manuscript, you will be asked to respond to the reviewer(s) comments. Please be as specific as possible in your response to all of the comments.

The editorial team at Brain Communications also aims to increase transparency in scientific publishing and to demonstrate the value that peer review brings to the scientific process. Please therefore consider whether you are willing to have reviewer comments on your paper and your response published as supplementary data with the paper, if it is accepted. This is entirely optional and will only be done if both the authors and reviewers agree. When submitting your revision you will have the opportunity to update the answer you entered previously.

IMPORTANT: Your original files are available to you when you upload your revised manuscript. Please delete any redundant files before completing the submission.

Because we are trying to facilitate timely publication of manuscripts submitted to Brain Communications, we would appreciate your revised manuscript as soon as possible. If it is not possible for you to resubmit within 3 months, the option to submit a revision will expire, and we would then consider your revised paper as a new submission. However, if you need a little more time, please get in touch and we can extend this deadline for you.

Once again, thank you for submitting your manuscript to Brain Communications and I look forward to receiving your revision.

Best wishes

Dr. Rakez Kayed
Associate Editor, Brain Communications

Professor Tara Spires-Jones
Founding Editor, Brain Communications

Reviewer(s)' Comments to Author:

Reviewer: 1 (Nicha Puangmalai)

Comments to the Author
Progranulin (PGRN) is implicated in the pathogenesis of a variety of diseases; however, it is most strongly tied to frontotemporal dementia. In this study, Du et al. demonstrated the role of sortilin and prosaposin (PSAP) in PGRN lysosome trafficking and processing in vivo. Authors generated mice deficient in PSAP and/or sortilin and analyzed PGRN lysosome localization, levels of granulin peptides and PGRN in serum.
Authors show that PSAP and sortilin are the only two pathways involved in PGRN lysosome trafficking in neurons, while PSAP and sortilin independent PGRN lysosome trafficking mechanism exists in microglia.

Overall, there are some minor concerns/suggestions about the experiments listed below.
1. Please mention the number of animals used in this study.
2. Biochemical analysis from neuronal and microglia enriched fractions from mice different ages should confirm the immunofluorescence results in figure 1 and 2
3. Microglia PGRN levels and PGRN/LAMP1 colocalization analysis should present in figure 4.
4. It is good to have results from primary glial culture along with results in BV2 cells.
5. Please check typo error in Introduction.

Reviewer: 2

Comments to the Author
The authors present the potential mechanisms for lysosomal trafficking of PGRN via sortilin and PSAP binding. The work mainly expands on the previous findings by the same authors using mice models. They argue that in mice with PSAP and sortilin ablation, the levels of circulating PGRN is increased significantly along with increase in the levels of Grn peptides (cleavage products of PGRN), and significant decrease in the ratio of Grns to PGRN. Interestingly, the authors also argue that ablation of Sort and PSAP does not affect the lysosomal trafficking of PGRN in microglia and this trafficking is independent of Sort/PSAP pathway. The study is straightforward, simple, and well-designed. Following are some comments/questions that will further improve the study –
1. Can authors elaborate why the ratio of GRNS/PGRN is low in cortical lysates of PSAP-/- mice even as the levels of granulins are high? Same question in case of Sot-/- PSAP-/- mice.
2. Since there is a decline in the Grns to PGRN ratio in PSAP -/- and sort-/- PSAP-/- mice, did the authors measure the ratio of Grns/PGRN in WT mice? Or is this ratio available in literature. If yes, can authors elaborate and compare with the Grns/PGRN ratio in PSAP and Sort-PSAP knockouts?
3. Did the authors observe in phenotypic/behavioral changes in the mice with sortilin ablation? Since several clinical trials are looking at blocking sortilin receptors to increase circulation levels of PGRN in FTLD patients, it is important to see any behavioral changes in mice with sortilin ablation?
4. Can authors quantify the 'slight decrease' in the ratio of Grns/PGRN in Sort-/- mice (line 12, page 8)?

Minor comments –
1. Rephrase line 38, page 8
2. Quite a few places, the citation format doesn't match the requirements, for example, line 42, page 3.

Comments from the Scientific Editor:

-For all the plots, please, provide details of the experimental unit (n= mice, cells, wells, plate downs, etc)) for each group analysed in these plots, and since the number is <10 show the single data-points. Please note our policy on pseudoreplication, we do not accept analyses where individual cells are the experimental unit when there is likely biological variability is between animals, culture replicates, etc. Please see instructions for authors for more details. Fort his type of data, we require either that a mean/median be taken per animal/culture replicate followed by statistical analyses or that more sophisticated analyses are performed such as mixed effects modelling which can account for random variables.

- please, provide Full- size and uncropped blots/gels as Supplementary material.

-Please supply a graphical abstract. A graphical abstract is a visual representation of the central finding or methodology of the paper. It will be displayed in the table of contents. Authors should strive to make the graphical abstract informative, interesting, visually appealing, and straightforward (no caption needed, no abbreviations except gene names). Please upload as a separate high resolution image file

(tiff preferred). The technical requirements for the graphical abstracts are:
--size of the submitted image: 1200 pixels square at 300dpi
--font of the text: Arial 12-16 point (smaller fonts will not be legible online)

-Please add an Abbreviated Summary (up to 50 words) of your paper that captures the main purpose and conclusions of the work. The summary should state the main results written in the third person (‘[1st author surname] et al. report that …’) and conclusion. This summary will be used in the contents list online and should not contain any abbreviations other than those on the accepted abbreviations list and accepted gene/protein names, making it accessible to the non-specialist. Please upload it as a Word document. Allowed abbreviations: AIDS; ANOVA; AMPA; ATP; A,T,C,G; CNS; CSF; CT; DNA; ECG; EEG; EMG; GABA; HIV; MRI; NMDA; PET; PCR; RNA.

-Please include a paragraph about data availability in the 'Materials and Methods' section. We strongly encourage data sharing either as supplementary information or uploads to repositories which you can link in the paper.

- To comply with our ethical standards, the appropriate checklist must be included (see Reporting Guideline section within 'Instructions to authors').
